# Plastic Germination, Temporal Niche Partitioning and Emergent Assortative Mating in Annual Plants

**DOI:** 10.1111/ele.70346

**Published:** 2026-02-19

**Authors:** Max Schmid, Katja Tielbörger, Amaël Daval, Charles Mullon

**Affiliations:** ^1^ Institute of Evolution and Ecology University of Tübingen Tübingen Germany; ^2^ Department of Ecology and Evolution University of Lausanne Lausanne Switzerland

**Keywords:** annual plants, ecological speciation, genetic linkage, phenotypic plasticity, predictive germination, storage effect

## Abstract

Temporal fluctuations in the environment can promote coexistence via the storage effect, where competing variants are buffered during unfavourable years. In annual plants, this can arise from seed dormancy: seeds remain in the seed bank across years and germinate under suitable conditions. Here, we investigate how plasticity in germination timing (where seeds use environmental cues to adjust when they germinate) affects genetic diversification and ecological speciation. Using eco‐evolutionary models, we show that adaptive plasticity readily evolves via genetic associations between germination and fecundity traits, allowing seeds to germinate preferentially in years favourable for reproduction. This enhances temporal niche partitioning and promotes divergence into specialised morphs. Because these morphs germinate in different years, plasticity generates temporal assortative mating and maintains trait associations even without genetic linkage. Our results show that adaptive plasticity and genetic diversification can interact synergistically: predictive germination not only buffers fluctuations but also drives the evolution of biodiversity.

## Graphical abstract

Temporal environmental fluctuations can drive genetic diversity and species coexistence via the storage effect. Using eco‐evolutionary models of annual plants, we show that adaptive plasticity in seed germination readily evolves and promotes genetic diversification by facilitating temporal niche partitioning, and in sexual populations can initiate ecological speciation by generating temporal assortative mating. These results highlight how adaptive plasticity and genetic diversification can interact synergistically to enhance biodiversity.

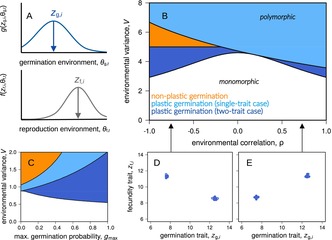

## 1 Introduction

Most communities experience fluctuations in environmental conditions, with year‐to‐year variation in temperature, rainfall or disturbances such as fires or floods (Grant et al. 2017; Ummenhofer and Meehl 2017; He et al. 2019; Tielbörger et al. 2014; Pausas and Lamont 2022). Such temporal variability can promote biodiversity by maintaining genetic and/or phenotypic variation in two main ways. First, individuals can evolve to respond plastically to prevailing conditions, resulting in environmentally induced phenotypes (polyphenism; Woltereck 1909; Bradshaw 1965; Scheiner 1993; Schlichting 1986; Via et al. 1995). The evolution of adaptive plasticity requires reliable cues that predict the selective environment (Moran 1992; Tufto 2000; Bonamour et al. 2019), as well as sufficiently flexible developmental programs (DeWitt et al. 1998; Lande 2014; Dupont et al. 2024). Second, populations can evolve genetic polymorphisms, or species within communities can diverge via character displacement, leading to temporal niche partitioning. Here, different variants (alleles or species) specialize on contrasting yearly conditions (Chesson and Warner 1981; Ellner and Hairston 1994; Yamamichi et al. 2023). The evolution of temporal niche partitioning requires a mechanism buffering variants against selection in unfavorable years, known as the temporal storage effect (P. L. Chesson 1983; Svardal et al. 2015; Yamamichi et al. 2023). Determining how these processes shape genetic and phenotypic variation is essential for understanding biodiversity and its maintenance in temporally variable environments.

Adaptive plasticity and genetic polymorphism are typically viewed as alternatives but these processes may also interact (e.g., West‐Eberhard 1989; Crispo 2008; Conover et al. 2009; Ghalambor et al. 2015; Gulisija et al. 2016; Schmid and Guillaume 2017; Sommer 2020). One context where this interaction might be particularly relevant is temporal niche partitioning in annual plants. Under interannual fluctuations, niche partitioning readily evolves when seeds exhibit between‐year dormancy, whereby a fraction remains dormant for multiple years before germinating (P. L. Chesson 1994; Ellner and Hairston 1994; P. Chesson 2000; Barabás et al. 2018; P. Chesson 2020; Johnson and Hastings 2022), generating the necessary storage effect. Seed germination itself can show adaptive plasticity, with seeds germinating preferentially under conditions predicting favourable subsequent growth and reproduction (e.g., Angert et al. 2009; Donohue et al. 2010; Cohen 1967). Such predictive germination (Venable and Lawlor 1980; Pake and Venable 1996) can reinforce character displacement, causing different species to germinate in different years, thereby facilitating coexistence via the storage effect (Kortessis and Chesson 2021). However, existing theory has so far treated predictive germination mainly as a mechanism maintaining differences among already diverged species (Venable et al. 1993; Snyder and Adler 2011; Mathias and Chesson 2013; Kortessis and Chesson 2021). What remains unclear is whether the same process can also create diversity in the first place, by initiating genetic diversification and ecological speciation within initially homogeneous populations.

Here, we extend current theory to investigate whether the evolution of predictive germination facilitates the emergence and divergence of genetic morphs within annual plant species. We show that predictive germination enhances the conditions under which temporal niche partitioning evolves. We further explore evolutionary constraints imposed by sexual reproduction and recombination, revealing that adaptive divergence in germination timing generates temporal assortative mating, thus setting the stage for ecological speciation.

## 2 Model

### 2.1 Life Cycle and Traits

We model a large population of annual plants that shows between‐year seed dormancy (though this model can equally apply to other taxa, such as *Daphnia* with resting eggs or fungi with dormant spores). Seeds may remain dormant in the seed bank for multiple years, while plants that germinate are short‐lived and die after reproduction.

The population may experience environmental conditions during germination and reproduction that vary between years (e.g., fluctuations in humidity, temperature or herbivore density). We represent these fluctuations with two random variables per year t: $\theta_{g,t}$, describing the environment during the germination period, and $\theta_{f,t}$, describing the environment later in the year during reproduction. These conditions influence germination and reproduction through interactions with specific plant traits, as detailed below.

#### 2.1.1 Seed Germination

At the beginning of each growing season, seeds in the seed bank either remain dormant or germinate. The probability that a seed indexed i, characterised by its germination trait $z_{g,i}$, germinates in year t depends on the early‐year environment $\theta_{g,t}$ (e.g., temperature at the time of rainfall, see Baskin et al. 1993) as follows:

(1) $$g\left(z_{g,i},\theta_{g,t}\right)=g_{\max}\;\mathrm{Exp}\left[-\frac{{\left(z_{g,i}-\theta_{g,t}\right)}^{2}}{2\;\sigma_{g}^{2}}\right]$$

**FIGURE 1:**
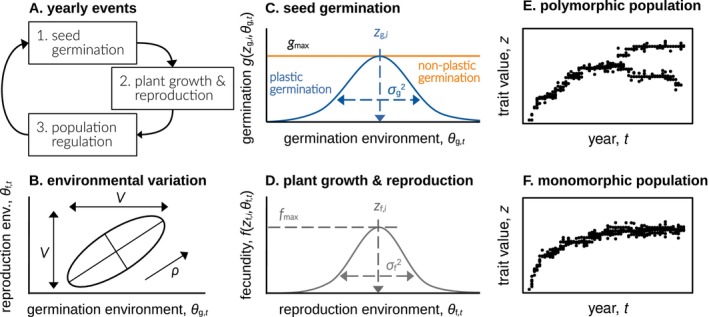
Graph A shows the yearly life‐cycle events, where a fraction of seeds germinates, reproduces, and the plant population then experiences density regulation. The ellipsoid in graph B illustrates the environmental distribution where environmental conditions during germination $\theta_{g,t}$ (*x*‐axis) and reproduction $\theta_{f,t}$ (*y*‐axis) are drawn from a bivariate normal distribution, each year anew. Both environmental conditions show between‐year variance V and within‐year environmental correlation ρ. Graph C contrasts plastic seed germination (blue line) with non‐plastic seed germination (orange line, where $\sigma_{g}^{2}\to \infty$). Graph D shows how the individual fecundity changes with the match between $\theta_{f,t}$ and the individual fecundity trait $z_{f,i}$. The evolutionary dynamics in $z_{g}$ and $z_{f}$ then could either lead to a polymorphic plant population under disruptive selection (E), or to a monomorphic population with stabilising selection (F).

Equation 1 indicates that a seed germinates with a maximum probability, $0<g_{\max}\leq 1$, when its trait matches environmental conditions (i.e., when $\theta_{g,t}=z_{g,i}$; Figure 1C). In this view, trait $z_{g,i}$ could be seen as a physiological or morphological seed character that regulates the preferred environment for germination. Germination probability declines with increasing mismatch between a seed's trait and the environment, at a rate determined by $\sigma_{g}^{2}>0$.

Equation 1 sets a reaction norm describing how seed germination responds to the environment with the parameter $\sigma_{g}^{2}$ tuning the degree of germination plasticity. A small $\sigma_{g}^{2}$ corresponds to high plasticity, meaning germination probabilities change strongly in response to environmental variation (blue line in Figure 1C; e.g., see Simons 2014, for empirical data supporting bell‐shaped germination functions in response to temperature). In contrast, large $\sigma_{g}^{2}$ corresponds to low plasticity, meaning germination probabilities remain relatively constant across a broad range of environmental conditions. In the limiting case ($\sigma_{g}^{2}\to \infty$), germination becomes completely independent of the environment (non‐plastic), reducing the reaction norm to a horizontal line with intercept $g_{\max}$ (orange line in Figure 1C; e.g., see Ellner and Hairston 1994).

#### 2.1.2 Plant Growth and Reproduction

After germination, plants grow and reproduce. We assume that the fecundity of plant i, characterised by its fecundity trait $z_{f,i}$, in response to the reproduction environment $\theta_{f,t}$ (e.g., temperature during flowering), is given by.

(2) $$f\left(z_{f,i},\theta_{f,t}\right)=f_{\max}\;\mathrm{Exp}\left[-\frac{{\left(z_{f,i}-\theta_{f,t}\right)}^{2}}{2\;\sigma_{f}^{2}}\right]$$

where maximum fecundity $f_{\max}$ is reached when the fecundity trait matches environmental conditions during reproduction (i.e., when $z_{f,i}=\theta_{f,t}$; Figure 1D). Trait $z_{f,i}$ could be seen as a quantitative trait in germinated plants that governs the preferred environment for reproduction (like root morphology or stomata density). Fecundity declines with increasing mismatch between trait and environment at a rate determined by $\sigma_{f}^{2}>0$. This parameter $\sigma_{f}^{2}$ measures the breadth of the fecundity niche, with large $\sigma_{f}^{2}$ indicating a broad niche (plants achieve high fecundity across a wide range of conditions) and small $\sigma_{f}^{2}$ indicating a narrow niche (high fecundity is limited to specific environmental conditions). Equation 2 represents a classical assumption in many theoretical models of adaptation to environmental variables (e.g., Via and Lande 1985; Day 2000; Kisdi 2002; Chevin and Lande 2011; Svardal et al. 2015; Miller and Klausmeier 2017; Yamamichi et al. 2019; Ohtsuki et al. 2020; Orive et al. 2023; Saltini et al. 2023).

#### 2.1.3 Regulation

After reproduction, all germinated plants die. Seeds that did not germinate remain dormant, surviving to the next year with probability s and dying with probability 1−s. Seed‐bank slots are freed when seeds germinate (and thus leave the seed bank) or when dormant seeds die. Newly produced seeds then compete for these vacant slots. We assume the seed‐bank size is constant, with N seeds present at the start of each year (with N large), and that recruitment into vacant slots is random with respect to trait values, following a lottery model (Chesson and Warner 1981).

The assumption of a constant seed‐bank size, which greatly facilitates our mathematical analysis, may seem contrived and biologically unrealistic. However, simulations where we relax this assumption and let the size of the seed bank fluctuate year‐to‐year (in a similar way to Kortessis and Chesson 2021) indicate that it has little effect on our model's results (see Appendix D.4).

### 2.2 Environmental Heterogeneity and Genetic Constraints

We assume environmental conditions during germination ($\theta_{g,t}$) and reproduction ($\theta_{f,t}$) fluctuate randomly among years around the same long‐term mean and with equal variance ($E\left[\theta_{g,t}\right]=E\left[\theta_{f,t}\right]=\bar{\theta }$ and $V\left[\theta_{g,t}\right]=V\left[\theta_{f,t}\right]=V$ for all t; Supporting Information Appendix A). Conditions during germination and reproduction within the same year may be correlated ($\text{Corr}\left[\theta_{g,t},\theta_{f,t}\right]$ = ρ for all t; Figure 1B; e.g., see Kortessis and Chesson 2021). A positive within‐year correlation (ρ>0) implies predictive germination cues (e.g., dry germination periods predict dry reproductive periods), whereas a negative correlation (ρ<0) indicates an inverse relationship (e.g., dry germination periods predict wet reproductive periods). Weak correlations (ρ≈0) imply limited predictability between conditions at germination and reproduction, likely impeding the evolution of predictive germination.

We explore how genetic constraints interact with environmental correlations to influence adaptation and diversification. We consider the joint evolution of the germination ($z_{g}$) and fecundity ($z_{f}$) trait, which together determine environmental conditions at germination and subsequent reproduction. We compare two scenarios: (i) the *single‐trait case*, where germination and fecundity traits are genetically constrained to be identical ($z_{g,i}=z_{f,i}$; such that each individual maximises germination and subsequent fecundity under the same conditions), and (ii) the *two‐trait case*, where traits evolve independently (such that each individual may adapt to different conditions for germination and for reproduction). Comparing these scenarios allows examination of how genetic constraints influence the evolution of temporal niche partitioning under different degrees of environmental correlation.

### 2.3 Analyses

We aim to identify the ecological and genetic conditions under which germination and fecundity traits become polymorphic, thus leading to temporal niche partitioning. We use two complementary approaches.

First, we conduct evolutionary invasion analyses (Supporting Information B and C), assuming haploid, clonally reproducing plants. Each trait is encoded by a single locus evolving under the continuum‐of‐alleles model. Mutations are rare and have small phenotypic effects such that evolution occurs in two phases (Geritz et al. 1998). Initially, directional selection drives the population toward convergence‐stable trait values with the population remaining largely monomorphic. Subsequently, the population either remains monomorphic under stabilising selection (Figure 1F) or undergoes evolutionary branching, becoming genetically and phenotypically polymorphic under disruptive selection (Figure 1E). Our goal is to identify conditions favouring evolutionary branching.

Second, we use individual‐based simulations, relaxing key analytical assumptions by explicitly incorporating diploidy, sexual reproduction and varying genetic linkage between germination and fecundity loci. These simulations test the robustness of our analytical predictions and allow us to investigate how sexual reproduction and recombination influence the evolution of trait polymorphism and temporal niche partitioning (Supporting Information Appendix D). They also allow us to analyze the emergence of temporal assortative mating and its consequences for ecological speciation.

## 3 Results

### 3.1 Germination Plasticity Facilitates Polymorphism Under Positive Environmental Correlations and Genetic Constraints

We first investigate the *single‐trait case* where individuals achieve maximum germination and maximum reproduction at the same environmental condition (i.e., $z_{g,i}=z_{f,i}$ due to genetic constraints). As a baseline, we compare our results to the classical scenario without plasticity ($\sigma_{g}^{2}\to \infty$; Figure 1C), for which polymorphism evolves under the condition $\left(1-g_{\max}\right)\mathrm{sV}>\sigma_{f}^{2}$ (orange areas in Figure; P. L. Chesson 1983; Ellner and Hairston 1994; Svardal et al. 2015). Polymorphism in this baseline scenario is thus favoured by high between‐year environmental variability (V), narrow fecundity niches (small $\sigma_{f}^{2}$), and extensive seed dormancy (small $g_{\max}$, large s).

**FIGURE 2:**
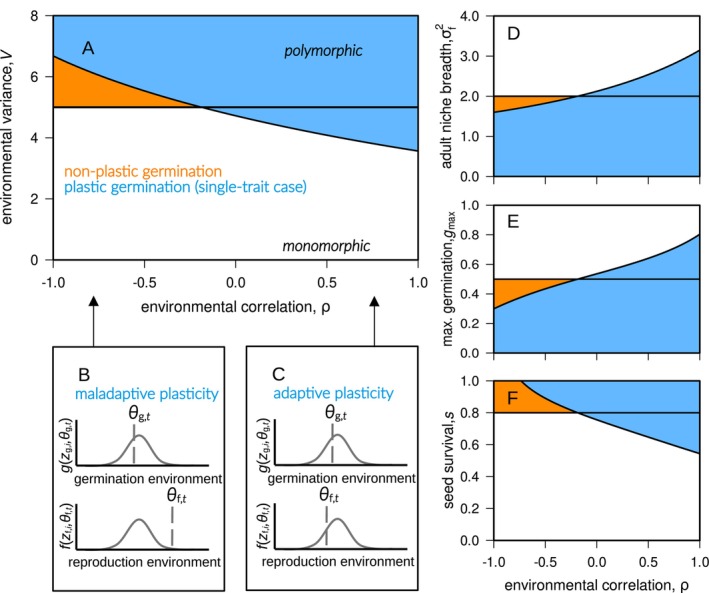
Graphs A, D–F present analytical results for the *single‐trait case* ($z_{g,i}$ = $z_{f,i}$) where plants evolve genetic/phenotypic polymorphism in the coloured areas, but stay monomorphic in the white areas. The case of plastic seed germination is presented in blue (when $\sigma_{g}^{2}$ = 20; according to equation B13 with components B16), the case of non‐plastic germination is shown in orange (when $\sigma_{g}^{2}\to \infty$; following $\left(1-g_{\max}\right)\mathrm{sV}>\sigma_{f}^{2}$). If not specified in graph B and D–F, the parameter values are: $g_{\max}$ = 0.5, s = 0.8, $\sigma_{f}^{2}$ = 2, V = 5, and ρ = 1. Graphs B and C conceptualise how environmental correlation leads to adaptive and maladaptive germination plasticity. With ρ<0 and large differences between $\theta_{g,t}$ and $\theta_{f,t}$ within years, a specialised plant morph ($z_{g}\neq \bar{\theta }$) might experience suitable conditions for germination, but detrimental conditions during reproduction (graph B). With ρ>0, environmental conditions do not change much within years ($\theta_{g,t}\approx \theta_{f,t}$) such that plants with $z_{g,i}=z_{f,i}$ could combine high germination probabilities with high fecundities.

When allowing plastic germination ($\sigma_{g}^{2}<\infty$), conditions for evolutionary branching are more involved mathematically (see equation B16 in Supporting Information Appendix B for an explicit analytical solution) but can be easily interpreted graphically (Figure 2). Plastic germination broadens conditions leading to polymorphism under positive within‐year environmental correlations (blue areas in Figure 2, ρ>0). Here, plasticity allows plants to germinate preferentially in years reliably favourable for subsequent reproduction; that is, plasticity in this case is adaptive (following Gotthard and Nylin 1995's conceptual distinction). For instance, plasticity evolves such that plants adapted to warm conditions germinate and reproduce predominantly in warm years, while those adapted to cooler conditions germinate predominantly in cooler years. By allowing plants specialised to distinct environmental conditions to reproduce mostly in separate years, plastic germination here reduces between‐morph competition and enhances temporal niche partitioning.

Conversely, plastic germination restricts polymorphism relative to the non‐plastic scenario under negative environmental correlations (blue areas in Figure 2, ρ<0). In this situation, the same environmental preference during germination and fecundity cannot simultaneously match contrasting environmental conditions within a year (i.e., $\theta_{g,t}\neq \theta_{f,t}$ when ρ→−1; see equation A11). This mismatch means seeds germinate into conditions detrimental for reproduction, so germination plasticity here is maladaptive (Figure 2B). For example, warm‐adapted plants germinate preferentially under warm conditions but then experience cold conditions during reproduction. Such mismatch in turn hinders temporal niche partitioning.

Overall, we see that adaptive germination plasticity promotes polymorphism while maladaptive plasticity opposes it. This parallels findings by Kortessis and Chesson (2021), who showed that predictive germination promotes divergence between differentiated species when reliable cues are present and when the germination schedule can be aligned to beneficial conditions for plant growth.

In the absence of environmental correlation within years (ρ=0), germination plasticity can either facilitate or oppose polymorphism relative to the non‐plastic scenario, depending on parameters (Figure 2, horizontal line at ρ=0; equation B19). Two opposing effects explain this result. On the one hand, increased plasticity (smaller $\sigma_{g}^{2}$) reduces the long‐term average germination probability, increasing seed dormancy and thus strengthening the storage effect, thereby favouring polymorphism. On the other hand, increased plasticity induces stabilising selection on the trait during germination when environmental variability (V) or seed survival (s) is limited (in fact, selection is stabilising if $\mathrm{sV}<\sigma_{g}^{2}$ when fecundity selection is weak, $\sigma_{f}^{2}\to \infty$). The interplay of these two effects determines whether germination plasticity promotes or inhibits polymorphism when ρ=0.

### 3.2 Temporal Niche Partitioning Under Positive and Negative Environmental Correlation When Germination and Fecundity Traits Evolve Independently

**FIGURE 3:**
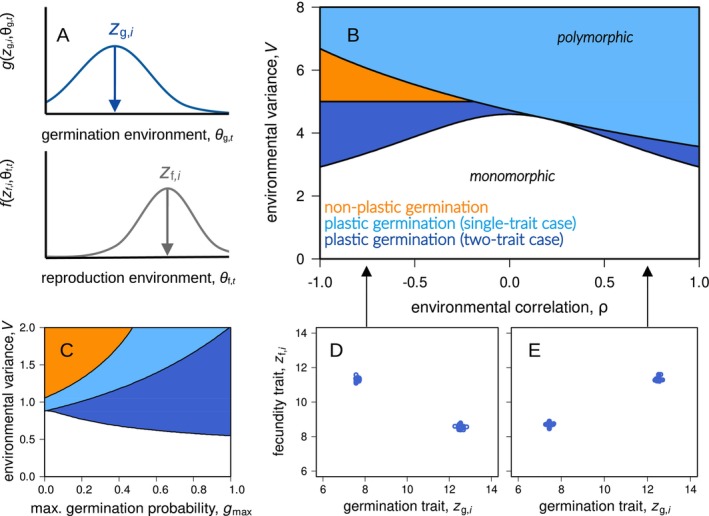
Graph A illustrates the *two‐trait case* where the germination trait and the fecundity trait could evolve independently and differ from each other within individuals (such that $z_{g,i}\neq z_{f,i}$). The analytical results for the two‐trait case in graphs B and C present conditions leading to trait polymorphism and niche partitioning (dark blue area), and conditions leading to monomorphic plant populations (white area; from the Hessian matrix C6 with components C17‐C19). Note that the orange and light blue colours in graph B and C mask the dark blue areas underneath. Graphs D, E illustrate how plants could diversify with negative (positive) environmental correlation by the evolution of negative (positive) covariance between individual $z_{g,i}$ and $z_{f,i}$ values. Parameter values for graphs (B, D, E) are: $g_{\max}$ = 0.5, s = 0.8, $\sigma_{f}^{2}$ = 2, $\sigma_{g}^{2}$ = 20, V = 5, with ρ=1 (graph E) and ρ = −1 (graph D). The parameters for (graph C) are: s = 0.95, $\sigma_{f}^{2}$ = 1, $\sigma_{g}^{2}$ = 5, V = 5 and ρ = 1.

We next allow germination and fecundity traits to evolve independently (*two‐trait case*). Allowing independent trait evolution broadens conditions leading to polymorphism, now occurring under both strongly positive and strongly negative environmental correlations within years (high absolute values of ρ, dark blue areas in Figure 3B). Under negative correlation (ρ<0), polymorphism emerges because germination and fecundity traits can evolve in opposite directions, each trait matching distinct environmental conditions within the same year. For example, some plants evolve to germinate preferentially under cool conditions but reproduce best under warm conditions, while others evolve the opposite pattern. This generates negative covariance between germination and fecundity traits ($z_{g,i}$ and $z_{f,i}$; Figure 3D, see also equation C19 in Supporting Information Appendix C for the explicit analytical expression). Conversely, under positive environmental correlation (ρ>0), covariance between germination and fecundity traits becomes positive, aligning germination timing with conditions beneficial for reproduction (Figure 3E). Germination plasticity is thus adaptive at both positive and negative correlation, enhancing temporal niche partitioning whenever environmental correlations within years are sufficiently strong.

In the two‐trait scenario, high maximum germination probabilities (large $g_{\max}$) can facilitate polymorphism, particularly under strong environmental correlations (large ∣ρ∣) and high seed survival (s; dark blue area in Figure 3C). This contrasts with our single‐trait scenario and with earlier models (e.g., Wisnoski and Shoemaker 2022), where increased dormancy (lower $g_{\max}$) promotes polymorphism by enhancing the storage effect. This difference arises because independent evolution of the two traits enables the evolution of highly adaptive plasticity when ∣ρ∣ is large: specialised genotypes evolve to germinate almost exclusively in conditions that reliably predict high fecundity. As a consequence, increasing the maximum germination probability ($g_{\max}$) allows these specialised genotypes to express their traits more frequently when conditions are favourable. Seed survival during dormancy (s) modulates this effect by enabling seeds to persist through unfavourable periods, thus maintaining the specialised genotypes across years and sustaining the storage effect.

### 3.3 Plastic Seed Germination Drives Phenotypic Variation Within and Among Years

**FIGURE 4:**
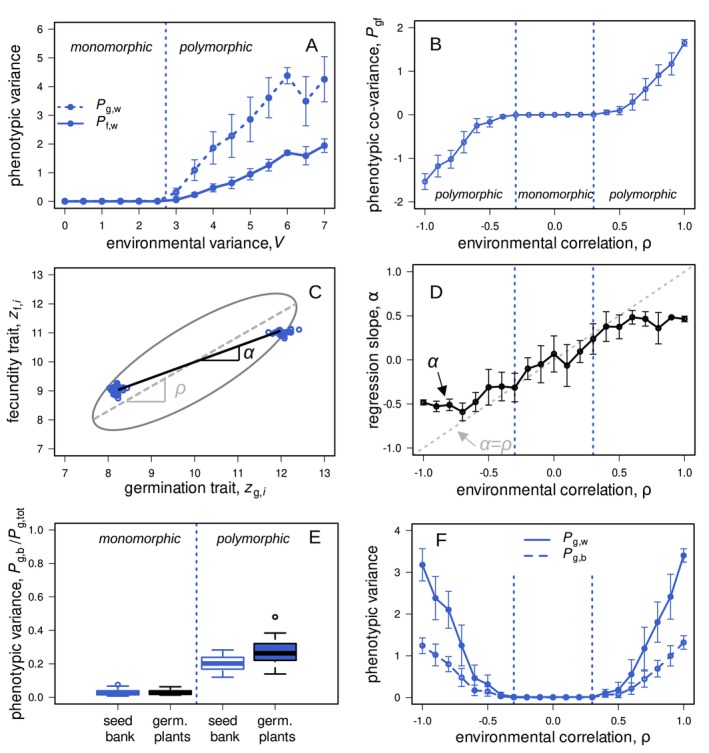
These graphs show simulation results for the two‐trait case with clonal reproduction in haploid plants. Graph A depicts within‐year phenotypic variance in the germination trait (dashed lines) and the fecundity trait (solid lines) as a function of interannual environmental variation V. The vertical dotted lines (—) present the threshold between stabilising and disruptive selection as predicted by the mathematical model (following the Hessian matrix C6 with components C17‐C19). Graph B illustrates the extent of trait covariance $P_{\mathrm{gf}}$ (see equation D7) as a function of environmental correlation, where polymorphism could evolve with positive and negative ρ. Graph C conceptualises the linear regression slope for the phenotypic distribution (α) and for the environmental distribution (ρ), where plants not always evolve α=ρ (graph D). Graph E shows simulation results of the between‐year phenotypic variance ($P_{g,b}/P_{g,\mathrm{tot}}$) for seeds in the seed bank and for germinated plants only. Graph F presents the between‐year phenotypic variance as a function of environmental correlation. The parameter values for graphs A‐F are: V = 4, $g_{\max}$ = 0.5, s = 0.8, $\sigma_{f}^{2}$ = 2, $\sigma_{g}^{2}$ = 10 (with ρ = 0.8 for graph A and C; and ρ = (0,1) for graph E). The whiskers in graphs A, B, D, F illustrate variation among replicates (covering one standard deviation).

**FIGURE 5:**
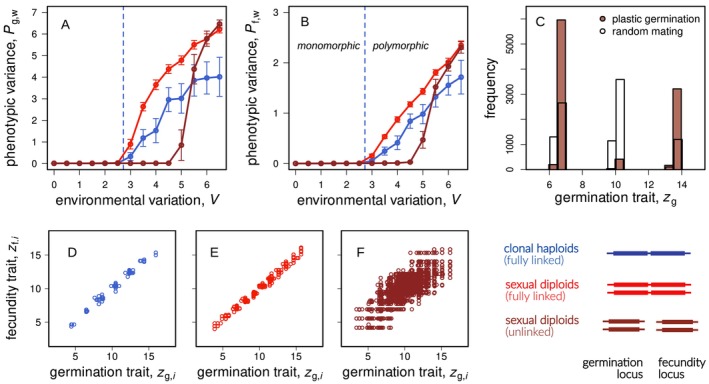
Graphs A‐F show simulation results for the *two‐trait case* with clonally reproducing haploid plants (in blue) and for outcrossing diploid plants with and without genetic linkage (light and dark red curves, respectively). The vertical dashed lines in graphs A and B present the analytical threshold between conditions leading to monomorphic and polymorphic plant populations (from the Hessian matrix C6 with components C17‐C19). The whiskers represent variation among replicates (spanning one standard deviation) and the parameter values for these simulations are: $g_{\max}$ = 0.5, s = 0.8, $\sigma_{f}^{2}$ = 2, $\sigma_{g}^{2}$ = 10, ρ = 1. The histograms in graph C present the phenotypic distribution at the end of a single simulation run with temporal assortative mating due to germination plasticity (dark red bars) and with random mating among all plant seeds (transparent bars). Temporal assortative mating leads to a reduced hybridization between the outer morphs relative to the case of random mating. Simulation parameters are the same as in graphs A‐B with V = 6. Graphs D–F show the phenotypic distribution of single simulation runs for varying genetic architectures with parameter values: $g_{\max}$ = 0.2, s = 0.98, $\sigma_{f}^{2}$ = 1, $\sigma_{g}^{2}$ = 1, V = 10, ρ = 0.9.

We next examined the phenotypic distributions at mutation‐selection‐drift equilibrium that arose in the individual‐based simulations. We measured phenotypic variance in the germination and fecundity traits ($P_{g,w}$ and $P_{f,w}$; see equation D5) in germinated plants. Conditions favouring polymorphism also led to greater phenotypic variance. Specifically, high environmental variability (V) and strong environmental correlation (∣ρ∣) resulted in increased phenotypic variance in both germination and fecundity traits (Figure 4A,F). This occurred because these conditions generated strong selection for coordinated trait combinations that were specialised to distinct yearly environmental conditions. When selection was sufficiently strong, more than two plant morphs could evolve and coexist (Figure 5D).

We further investigated how closely the phenotypic distributions of germinated plants matched the underlying environmental distributions (Figure 1B). First, we measured the phenotypic covariance ($P_{\mathrm{gf},w}$; see equation D7) between individual germination and fecundity traits ($z_{g,i}$ and $z_{f,i}$). The sign of this covariance consistently matched the sign of the environmental correlation ρ (Figure 4B), confirming our analytical predictions (equation C19 in Supporting Information Appendix C). Next, we compared the slope of the linear regression of germination on fecundity traits ($\alpha =P_{\mathrm{gf},w}/P_{g,w}$; Figure 4C) to the environmental correlation ρ. Though α and ρ were closely aligned, they did not match perfectly (i.e., α≠ρ; Figure 4D). Such deviations occurred because trait evolution depended not only on adaptation to environmental fluctuations, but also on reducing competition between specialised morphs. As a consequence, plants evolved phenotypic trait combinations that slightly deviated from a perfect match to environmental conditions. Similar results were found by Kortessis and Chesson (2021), who observed reduced predictive germination as a consequence of competition among plants.

We also measured between‐year phenotypic variance ($P_{g,b}$ and $P_{f,b}$; see equation D6). Polymorphic populations consistently exhibited higher between‐year variance (Figure 4E,F). Moreover, this variance was greater among germinated plants than among seeds in the seed bank (Figure 4E). This was because polymorphic populations could respond more strongly to changing selective pressures across years, causing rapid changes in morph frequencies that track environmental changes. Under plastic germination, only phenotypes well‐adapted to current environmental conditions germinated each year, even when the composition of the seed bank remained relatively stable. Thus, the phenotypic composition of germinated plants became uncoupled from that of the seed bank, leading to substantial year‐to‐year shifts. Between‐year variance among germinated plants accounted for up to approximately 30% of the total phenotypic variance (Figure 4E), indicating that a single‐year measurement captured only about 70% of the total long‐term phenotypic variance.

### 3.4 Outcrossing and the Rise of Temporal Assortative Mating

As a final extension, we examined the evolution of polymorphism in diploid plants with sexual reproduction. Here, germination and fecundity traits were controlled by separate genetic loci without physical linkage or pleiotropy, and mating was random. Under these conditions, polymorphism evolved under narrower environmental conditions compared to clonal reproduction (compare blue and dark red lines, Figure 5A,B). This was because recombination breaks down genetic associations between germination and fecundity traits, which were necessary for adaptation to correlated environments (ρ≠0). Consistent with this interpretation, when loci controlling germination and fecundity were completely linked, polymorphism evolved under conditions similar to those with clonal reproduction (light red lines, Figure 5A,B).

However, when environmental fluctuations were sufficiently strong, polymorphism emerged even without physical linkage between loci. This occurred through the evolution of temporal assortative mating driven by divergence in germination timing ($z_{g}$, dark red curves in Figure 5A,B). Plants with differentiated germination traits germinated preferentially in different years, thus reducing gene flow between differentiated morphs. This temporal segregation enabled genetic associations (linkage disequilibrium) between germination and fecundity traits to establish despite free recombination (Figure 5F). Temporal assortative mating was also reflected clearly in the phenotypic distribution of the population (Figure 5C). Under sexual reproduction, two highly differentiated morphs coexisted, along with an intermediate morph that resulted from mating between them. However, this intermediate morph occurred less frequently than would be expected if matings were entirely random (Figure 5C, red vs. white histograms).

Phenotypic variance was typically larger in polymorphic populations under sexual reproduction compared to clonal reproduction, in particular in the germination trait (Figure 5A, dark red curves exceed blue curves for large V). This was because divergence in the germination trait ($z_{g}$) was under additional selection in sexual populations as differentiated germination schedules led to temporal assortative mating, reducing the formation of maladapted hybrids (see Aubier et al. 2023, for a similar result). Additionally, sexual reproduction further led to a so‐called segregation variance (Slatkin and Lande 1994) which also contributed to greater $P_{g,w}$ and $P_{f,w}$ in our simulations with sexual reproduction (compare blue to red curves in Figure 5A,B).

## 4 Discussion

Our analyses indicate that adaptive plasticity in seed germination readily evolves and facilitates the emergence and maintenance of genetic diversity in annual plants under temporally fluctuating environments. Such predictive germination boosts the storage effect by allowing seeds to germinate preferentially in years favourable for growth and reproduction. This in turn promotes divergence among genetic morphs that can specialise to different yearly conditions and leads to temporal niche partitioning. In contrast, when genetic constraints lead to maladaptive plasticity, seeds often germinate into unfavourable conditions, opposing diversification. Our results thus extend existing theory on the storage effect (Venable et al. 1993; Snyder and Adler 2011; Kortessis and Chesson 2021), demonstrating that adaptive plasticity and genetic polymorphism can interact to reinforce biodiversity.

Predictive germination facilitates diversity by generating positive correlations between germination timing and favourable conditions for reproduction. When seeds reliably detect cues predicting good years, they can align their germination with environments best suited for growth and fecundity. Such alignment reduces competition among emerging genetic morphs, as each specializes on a distinct subset of years (Chesson and Warner 1981; Ellner and Hairston 1994). This interplay between germination and fecundity traits produces strong phenotypic covariance, further promoting niche partitioning and enabling stable coexistence (Kortessis and Chesson 2021). Adaptive germination plasticity thus enhances temporal niche differentiation beyond what is possible with divergence in a single trait.

Our results also suggest that predictive germination can promote reproductive isolation, thus setting the stage for ecological speciation. Divergence in germination timing generates temporal assortative mating, reducing gene flow among plant morphs specialised on different annual conditions. In that sense, the germination trait in our model can be seen as a ‘magic trait’, simultaneously controlling adaptation to the environment and facilitating reproductive isolation (Servedio et al. 2011). Such traits are particularly effective drivers of ecological speciation because they prevent genetic recombination and sexual reproduction from breaking down adaptive trait combinations (Dieckmann and Doebeli 1999; Gavrilets and Vose 2007). Even when loci controlling germination and fecundity traits are unlinked, temporal assortative mating allows trait covariance to emerge and persist, circumventing recombination's homogenising effects (Dieckmann and Doebeli 1999; Aubier et al. 2023). When temporal assortative mating via plastic germination is insufficient to override recombination, selection may favour tighter genetic linkage or pleiotropy between germination and post‐germination traits, potentially promoting ‘supergenes’ that further stabilise adaptive trait combinations (Sinervo and Svensson 2002). Alternatively, other mechanisms strengthening temporal assortative mating, such as genetic incompatibility systems, could enhance reproductive isolation, avoid the production of unfit hybrids and thus facilitate diversification.

Our findings contribute to a broader discussion on how phenotypic plasticity affects genetic diversification. Adaptive plasticity is often viewed as an alternative adaptation to genetic diversification, potentially limiting genetic divergence by enabling a single genotype to exploit multiple environments (Ghalambor et al. 2007; Pfennig et al. 2010; Turcotte and Levine 2016; Schmid and Guillaume 2017). Predictive germination provides an interesting exception, showing instead how plasticity can facilitate genetic diversification. This illustrates a more general mechanism whereby the existence of traits or traits' associations that allow genotypes to be preferentially expressed in contexts where they perform best favours diversification (Avila and Mullon 2023; Lehmann and Mullon 2025). Our arguments therefore also apply more broadly to life‐history plasticity, where adaptive transitions between life stages may allow populations to exploit distinct temporal niches more effectively than divergence in a single trait alone. Rather than being mutually exclusive, adaptive plasticity and genetic diversification can thus act synergistically to enhance biodiversity (see also West‐Eberhard 1989 for an alternative mechanism; Hendry 2016 and Sommer 2020 for a review).

Plastic seed germination evolves in our model, but it rarely yields perfect tracking of favourable years. Such limitations to plasticity are well known (DeWitt et al. 1998), and two arise directly under our assumptions. First, genetic architecture can prevent germination from aligning with favourable growth conditions: in the single‐trait case with negative within‐year correlation (ρ<0), the germination trait cannot diverge sufficiently from the fecundity trait, while in the two‐trait sexual case with weak or no linkage, recombination erodes the build‐up of adaptive covariance between germination and fecundity. Second, plastic responses are limited by imperfect environmental information: when germination cues are only weakly predictive of the subsequent reproductive environment, as is commonly assumed for natural systems (Hendry 2016), seeds inevitably germinate in some years that later prove unfavourable. In addition to these constraints, we did not model explicit costs of plasticity. Such costs have been documented (van Buskirk and Steiner 2009) and may arise from the machinery required for cue detection and processing (Scheiner et al. 2020). If present, these costs would reduce survival or fecundity of plastic genotypes and would therefore be expected to further limit the evolution of strong germination plasticity and contract the parameter range supporting temporal niche partitioning. Importantly, costs would change the quantitative scope of plasticity evolution, but not the qualitative mechanism by which cue‐based germination creates temporal niche differences and thereby promotes the storage effect.

Empirical studies provide support for our findings on predictive germination. For example, some long‐term studies of desert annual plants indicate associations between germination timing and conditions favourable for reproduction, such as specific rainfall or temperature cues (Venable et al. 1993; Pake and Venable 1996; Gremer et al. 2016). Also, germination fractions within a year seem to correlate with the expected competitive environment (Tielbörger and Valleriani 2005; Tielbörger and Prasse 2009). Other potentially relevant examples come from fire‐prone ecosystems, where germination cues such as smoke or heat may reliably predict beneficial growth conditions, including enhanced nutrient availability and reduced competition (Keeley and Pausas 2022). In these systems, germination traits correlate with adaptive post‐germination traits like high‐light tolerance or rapid post‐fire growth (Pausas and Lamont 2022). More broadly, a diverse set of environmental cues has been linked to predictive germination (including salinity and light cues, see table 2 in Donohue et al. 2010), and environmentally induced seed germination is associated with high diversification rates in several plant lineages (Willis et al. 2014).

Our findings also provide a potential explanation for the pronounced between‐year variation observed in some annual plant communities. In our model, predictive germination creates strong year‐to‐year changes in the phenotypic composition of germinated plants, despite a relatively stable composition of the underlying seed bank. Such between‐year fluctuations in community composition have indeed been reported from long‐term field surveys, with some species appearing abundantly in certain years and remaining virtually undetected in others (Pake and Venable 1996; Facelli et al. 2005; Levine et al. 2008; Tielbörger et al. 2014; Carrasco‐Puga et al. 2021). These dynamics could complicate biodiversity assessments because short‐term surveys may underestimate true diversity hidden in dormant seed banks (Carrasco‐Puga et al. 2021). Accurate evaluation of biodiversity under temporal fluctuations therefore calls either for long‐term monitoring data, comprehensive germination trials or genetic techniques such as environmental DNA analyses to fully capture community diversity.

Climate change could alter the ecological and evolutionary implications of predictive germination by modifying patterns of environmental predictability. As environmental cues may become less reliable with climate change (Kingsolver and Buckley 2018; Visser and Gienapp 2019; Edwards and Yang 2021), the capacity of seeds to match germination timing to optimal growth conditions may decline, reducing the effectiveness of temporal niche partitioning and potentially eroding biodiversity. In particular, increasingly erratic rainfall patterns, temperature shifts and altered disturbance regimes could disrupt previously stable associations between germination cues and favourable reproductive conditions. Such disruptions could disproportionately affect plant communities adapted to strongly correlated environmental fluctuations, possibly leading to declines in population sizes and shifts in species composition. Understanding how predictive germination strategies might respond to ongoing environmental change therefore emerges as a relevant question for future biodiversity dynamics.

Our model relies on several simplifying assumptions, and some modifications may be required to match particular systems. For instance, seed‐bank densities can vary substantially among years whereas we assume a constant size N. We relaxed this assumption in Supporting Information Appendix D.4 and still found that predictive germination strengthens the storage effect, consistent with Kortessis and Chesson (2021). In addition, while germination fractions in some systems, such as desert annuals, may increase monotonically with precipitation (e.g., see Lampei et al. 2017), we assume a bell‐shaped reaction norm; this affects quantitative details but not the core process by which predictive germination generates temporal niche differences. The model also omits parental effects (Tielbörger and Petrů 2010), which can contribute to temporal niche partitioning (Yamamichi and Hoso 2017); if adaptive, they would be expected to amplify the effects of predictive germination. Finally, germination and fecundity are often polygenic traits, whereas we modelled a one‐locus‐one‐trait architecture. Polygenic architectures may initially impede the build‐up of adaptive covariances between traits (e.g., Chebib and Guillaume 2022). However, if genetic modifiers are allowed to evolve, selection should favour a concentration of genetic effects that reduces recombination load, thereby facilitating the association between germination and fecundity traits.

To conclude, our study reveals that adaptive plasticity in seed germination promotes genetic diversification by facilitating temporal niche partitioning and reproductive isolation in temporally fluctuating environments. These results highlight that adaptive plasticity and genetic diversification are not mutually exclusive but instead can act together to enhance biodiversity. Understanding such interactions will help us better understand how natural populations might respond to future environmental change.

## Author Contributions

Max Schmid, Amaël Daval, Charles Mullon and Katja Tielbörger conceived the project. Max Schmid performed the mathematical analyses with input from Charles Mullon, and Max Schmid ran the simulations with contributions from Amaël Daval. Max Schmid wrote the first draft of the manuscript, Charles Mullon revised the draft. All authors contributed to the final version.

## Funding

This work was supported by SAGE, a Global Climate Centre, funded by the German Academic Exchange Service (DAAD).

## Conflicts of Interest

The authors declare no conflicts of interest.

## Supporting information


**Data S1:** ele70346‐sup‐0001‐supinfo.pdf.

## Data Availability

The *R* script, the simulation data, and the single graphs are available on figshare at https://doi.org/10.6084/m9.figshare.30196897 (Schmid et al. 2026).

## References

[ele70346-bib-0001] Angert, A. L. , T. E. Huxman , P. Chesson , and D. L. Venable . 2009. “Functional Tradeoffs Determine Species Coexistence via the Storage Effect.” Proceedings of the National Academy of Sciences 106, no. 28: 11641–11645. 10.1073/pnas.0904512106 PMC2710622 19571002

[ele70346-bib-0002] Aubier, T. G. , R. Bürger , and M. R. Servedio . 2023. “The Effectiveness of Pseudomagic Traits in Promoting Premating Isolation.” Proceedings of the Royal Society B: Biological Sciences 290: 20222108. 10.1098/rspb.2022.2108 PMC9993058 36883275

[ele70346-bib-0003] Avila, P. , and C. Mullon . 2023. “Evolutionary Game Theory and the Adaptive Dynamics Approach: Adaptation Where Individuals Interact.” Philosophical Transactions of the Royal Society B 378: 20210502. 10.1098/rstb.2021.0502 PMC10024992 36934752

[ele70346-bib-0004] Barabás, G. , R. D'Andrea , and S. M. Stump . 2018. “Chesson's Coexistence Theory.” Ecological Monographs 88, no. 3: 277–303.

[ele70346-bib-0005] Baskin, C. C. , P. L. Chesson , and J. M. Baskin . 1993. “Annual Seed Dormancy Cycles in Two Desert Winter Annuals.” Journal of Ecology 81, no. 3: 551–556.

[ele70346-bib-0006] Bonamour, S. , L.‐M. Chevin , A. Charmantier , and C. Teplitsky . 2019. “Phenotypic Plasticity in Response to Climate Change: The Importance of Cue Variation.” Philosophical Transactions of the Royal Society, B: Biological Sciences 374, no. 1768: 20180178. 10.1098/rstb.2018.0178 PMC6365871 30966957

[ele70346-bib-0007] Bradshaw, A. D. 1965. “Evolutionary Significance of Phenotypic Plasticity in Plants.” Advances in Genetics 13: 115–155.

[ele70346-bib-0008] Carrasco‐Puga, G. , F. P. Díaz , D. C. Soto , et al. 2021. “Revealing Hidden Plant Diversity in Arid Environments.” Ecography 44: 98–111.

[ele70346-bib-0009] Chebib, J. , and F. Guillaume . 2022. “The Relative Impact of Evolving Pleiotropy and Mutational Correlation on Trait Divergence.” Genetics 220, no. 1: iyab205. 34864966 10.1093/genetics/iyab205 PMC8733425

[ele70346-bib-0010] Chesson, P. 2000. “Mechanisms of Maintenance of Species Diversity.” Annual Review of Ecology and Systematics 31, no. 1: 343–366.

[ele70346-bib-0011] Chesson, P. 2020. “Chesson's Coexistence Theory.” Ecology 101, no. 11: e02851. 31351008 10.1002/ecy.2851

[ele70346-bib-0012] Chesson, P. L. 1983. “Coexistence of Competitors in a Stochastic Environment: The Storage Effect.” In Population Biology, edited by S. Levin , H. I. Freedman , and C. Strobeck , vol. 52, 188–198. Springer Berlin Heidelberg.

[ele70346-bib-0013] Chesson, P. L. 1994. “Multispecies Competition in Variable Environments.” Theoretical Population Biology 45: 227–276.

[ele70346-bib-0014] Chesson, P. L. , and R. R. Warner . 1981. “Environmental Variability Promotes Coexistence in Lottery Competitive Systems.” American Naturalist 117, no. 6: 923–943.

[ele70346-bib-0015] Chevin, L.‐M. , and R. Lande . 2011. “Adaptation to Marginal Habitats by Evolution of Increased Phenotypic Plasticity.” Journal of Evolutionary Biology 24, no. 7: 1462–1476. 21545421 10.1111/j.1420-9101.2011.02279.x

[ele70346-bib-0016] Cohen, D. 1967. “Optimizing Reproduction in a Randomly Varying Environment When a Correlation May Exist Between the Conditions at the Time a Choice Has to Be Made and the Subsequent Outcome.” Journal of Theoretical Biology 16, no. 1: 1–14. 6035758 10.1016/0022-5193(67)90050-1

[ele70346-bib-0017] Conover, D. O. , T. A. Duffy , and L. A. Hice . 2009. “The Covariance Between Genetic and Environmental Influences Across Ecological Gradients: Reassessing the Evolutionary Significance of Countergradient and Cogradient Variation.” Annals of the New York Academy of Sciences 1168: 100–129. 19566705 10.1111/j.1749-6632.2009.04575.x

[ele70346-bib-0018] Crispo, E. 2008. “Modifying Effects of Phenotypic Plasticity on Interactions Among Natural Selection, Adaptation and Gene Flow.” Journal of Evolutionary Biology 21, no. 6: 1460–1469. 18681916 10.1111/j.1420-9101.2008.01592.x

[ele70346-bib-0019] Day, T. 2000. “Competition and the Effect of Spatial Resource Heterogeneity on Evolutionary Diversification.” American Naturalist 155, no. 6: 790–803. 10.1086/303356 10805644

[ele70346-bib-0020] DeWitt, T. J. , A. Sih , and D. S. Wilson . 1998. “Costs and Limits of Phenotypic Plasticity.” Trends in Ecology & Evolution 13, no. 2: 77–81. 21238209 10.1016/s0169-5347(97)01274-3

[ele70346-bib-0021] Dieckmann, U. , and M. Doebeli . 1999. “On the Origin of Species by Sympatric Speciation.” Nature 400, no. 6742: 354–357. 10432112 10.1038/22521

[ele70346-bib-0022] Donohue, K. , R. Rubio De Casas , L. Burghardt , K. Kovach , and C. G. Willis . 2010. “Germination, Postgermination Adaptation, and Species Ecological Ranges.” Annual Review of Ecology, Evolution, and Systematics 41, no. 1: 293–319.

[ele70346-bib-0023] Dupont, L. , M. Thierry , L. Zinger , D. Legrand , and S. Jacob . 2024. “Beyond Reaction Norms: The Temporal Dynamics of Phenotypic Plasticity.” Trends in Ecology & Evolution 39, no. 1: 41–51. 37718228 10.1016/j.tree.2023.08.014

[ele70346-bib-0024] Edwards, C. B. , and L. H. Yang . 2021. “Evolved Phenological Cueing Strategies Show Variable Responses to Climate Change.” American Naturalist 197, no. 1: E1–E16.

[ele70346-bib-0025] Ellner, S. P. , and N. G. Hairston . 1994. “Role of Overlapping Generations in Maintaining Genetic Variation in a Fluctuating Environment.” American Naturalist 143, no. 3: 403–417.

[ele70346-bib-0026] Facelli, J. M. , P. Chesson , and N. Barnes . 2005. “Differences in Seed Biology of Annual Plants in Arid Lands: A Key Ingredient of the Storage Effect.” Ecology 86, no. 11: 2998–3006.

[ele70346-bib-0027] Gavrilets, S. , and A. Vose . 2007. “Case Studies and Mathematical Models of Ecological Speciation. 2. Palms on an Oceanic Island.” Molecular Ecology 16, no. 14: 2910–2921. 17614906 10.1111/j.1365-294X.2007.03304.x

[ele70346-bib-0028] Geritz, S. A. H. , E. Kisdi , G. Meszéna , and J. A. J. Metz . 1998. “Evolutionary Singular Strategies and the Adaptive Growth and Branching of the Evolutionary Tree.” Evolutionary Ecology 12: 35–57.

[ele70346-bib-0029] Ghalambor, C. K. , K. L. Hoke , E. W. Ruell , E. K. Fischer , D. N. Reznick , and K. A. Hughes . 2015. “Non‐Adaptive Plasticity Potentiates Rapid Adaptive Evolution of Gene Expression in Nature.” Nature 525: 372–375. 26331546 10.1038/nature15256

[ele70346-bib-0030] Ghalambor, C. K. , J. K. McKay , S. P. Carroll , and D. N. Reznick . 2007. “Adaptive Versus Non‐Adaptive Phenotypic Plasticity and the Potential for Contemporary Adaptation in New Environments.” Functional Ecology 21: 394–407.

[ele70346-bib-0031] Gotthard, K. , and S. Nylin . 1995. “Adaptive Plasticity and Plasticity as an Adaptation: A Selective Review of Plasticity in Animal Morphology and Life History.” Oikos 74, no. 1: 3–17.

[ele70346-bib-0032] Grant, P. R. , B. R. Grant , R. B. Huey , M. T. J. Johnson , A. H. Knoll , and J. Schmitt . 2017. “Evolution Caused by Extreme Events.” Philosophical Transactions of the Royal Society, B: Biological Sciences 372, no. 1723: 20160146. 10.1098/rstb.2016.0146 PMC5434096 28483875

[ele70346-bib-0033] Gremer, J. R. , S. Kimball , and D. L. Venable . 2016. “Within‐and Among‐Year Germination in Sonoran Desert Winter Annuals: Bet Hedging and Predictive Germination in a Variable Environment.” Ecology Letters 19, no. 10: 1209–1218. 27515951 10.1111/ele.12655

[ele70346-bib-0034] Gulisija, D. , Y. Kim , and J. B. Plotkin . 2016. “Phenotypic Plasticity Promotes Balanced Polymorphism in Periodic Environments by a Genomic Storage Effect.” Genetics 202, no. 4: 1437–1448. 26857626 10.1534/genetics.115.185702 PMC4905538

[ele70346-bib-0035] He, T. , B. B. Lamont , and J. G. Pausas . 2019. “Fire as a Key Driver of Earth's Biodiversity.” Biological Reviews 94, no. 6: 1983–2010. 31298472 10.1111/brv.12544

[ele70346-bib-0036] Hendry, A. P. 2016. “Key Questions on the Role of Phenotypic Plasticity in Eco‐Evolutionary Dynamics.” Journal of Heredity 107: 25–41. 26297912 10.1093/jhered/esv060

[ele70346-bib-0037] Johnson, E. C. , and A. Hastings . 2022. “Towards a Heuristic Understanding of the Storage Effect.” Ecology Letters 25, no. 11: 2347–2358. 36181717 10.1111/ele.14112

[ele70346-bib-0038] Keeley, J. E. , and J. G. Pausas . 2022. “Evolutionary Ecology of Fire.” Annual Review of Ecology, Evolution, and Systematics 53, no. 1: 203–225.

[ele70346-bib-0039] Kingsolver, J. G. , and L. B. Buckley . 2018. “How Do Phenology, Plasticity and Evolution Determine the Fitness Consequences of Climate Change for Montane Butterflies?” Evolutionary Applications 11: 1231–1244. 30151036 10.1111/eva.12618 PMC6099808

[ele70346-bib-0040] Kisdi, E. 2002. “Dispersal: Risk Spreading Versus Local Adaptation.” American Naturalist 159, no. 6: 579–596. 10.1086/339989 18707383

[ele70346-bib-0041] Kortessis, N. , and P. Chesson . 2021. “Character Displacement in the Presence of Multiple Trait Differences: Evolution of the Storage Effect in Germination and Growth.” Theoretical Population Biology 140: 54–66. 34058244 10.1016/j.tpb.2021.05.003

[ele70346-bib-0042] Lampei, C. , J. Metz , and K. Tielbörger . 2017. “Clinal Population Divergence in an Adaptive Parental Environmental Effect That Adjusts Seed Banking.” New Phytologist 214: 1230–1244. 28152187 10.1111/nph.14436

[ele70346-bib-0043] Lande, R. 2014. “Evolution of Phenotypic Plasticity and Environmental Tolerance of a Labile Quantitative Character in a Fluctuating Environment.” Journal of Evolutionary Biology 27, no. 5: 866–875. 24724972 10.1111/jeb.12360

[ele70346-bib-0044] Lehmann, L. , and C. Mullon . 2025. “Evolution of Quantitative Traits: Exploring the Ecological, Social and Genetic Bases of Adaptive Polymorphism.” bioRxiv: 2025.06.23.661088. 10.1016/j.jtbi.2026.112545 42398702

[ele70346-bib-0045] Levine, J. M. , K. A. McEachern , and C. Cowan . 2008. “Rainfall Effects on Rare Annual Plants.” Journal of Ecology 96, no. 4: 795–806.

[ele70346-bib-0046] Mathias, A. , and P. Chesson . 2013. “Coexistence and Evolutionary Dynamics Mediated by Seasonal Environmental Variation in Annual Plant Communities.” Theoretical Population Biology 84: 56–71. 23287702 10.1016/j.tpb.2012.11.009

[ele70346-bib-0047] Miller, E. T. , and C. A. Klausmeier . 2017. “Evolutionary Stability of Coexistence due to the Storage Effect in a Two‐Season Model.” Theoretical Ecology 10, no. 1: 91–103.

[ele70346-bib-0048] Moran, N. A. 1992. “The Evolutionary Maintenance of Alternative Phenotypes.” American Naturalist 139, no. 5: 971–989.

[ele70346-bib-0049] Ohtsuki, H. , C. Rueffler , J. Y. Wakano , K. Parvinen , and L. Lehmann . 2020. “The Components of Directional and Disruptive Selection in Heterogeneous Group‐Structured Populations.” Journal of Theoretical Biology 507: 110449. 32814071 10.1016/j.jtbi.2020.110449

[ele70346-bib-0050] Orive, M. E. , M. Barfield , and R. D. Holt . 2023. “Partial Clonality Expands the Opportunity for Spatial Adaptation.” American Naturalist 202, no. 5: 681–698. 10.1086/726335 37963114

[ele70346-bib-0051] Pake, C. E. , and D. L. Venable . 1996. “Seed Banks in Desert Annuals: Implications for Persistence and Coexistence in Variable Environments.” Ecology 77, no. 5: 1427–1435.

[ele70346-bib-0052] Pausas, J. G. , and B. B. Lamont . 2022. “Fire‐Released Seed Dormancy ‐ a Global Synthesis.” Biological Reviews 97, no. 4: 1612–1639. 35384243 10.1111/brv.12855 PMC9540907

[ele70346-bib-0053] Pfennig, D. W. , M. A. Wund , E. C. Snell‐Rood , T. Cruickshank , C. D. Schlichting , and A. P. Moczek . 2010. “Phenotypic Plasticity's Impacts on Diversification and Speciation.” Trends in Ecology & Evolution 25, no. 8: 459–467. 20557976 10.1016/j.tree.2010.05.006

[ele70346-bib-0054] Saltini, M. , P. Vasconcelos , and C. Rueffler . 2023. “Complex Life Cycles Drive Community Assembly Through Immigration and Adaptive Diversification.” Ecology Letters 26: 1084–1094. 37125448 10.1111/ele.14216

[ele70346-bib-0055] Scheiner, S. M. 1993. “Genetics and Evolution of Phenotypic Plasticity.” Annual Review of Ecology and Systematics 24: 35–68.

[ele70346-bib-0056] Scheiner, S. M. , M. Barfield , and R. D. Holt . 2020. “The Genetics of Phenotypic Plasticity. XVII. Response to Climate Change.” Evolutionary Applications 13, no. 2: 388–399. 31993084 10.1111/eva.12876 PMC6976953

[ele70346-bib-0057] Schlichting, C. D. 1986. “The Evolution of Phenotypic Plasticity in Plants.” Annual Review of Ecology and Systematics 17, no. 1: 667–693.

[ele70346-bib-0058] Schmid, M. , and F. Guillaume . 2017. “The Role of Phenotypic Plasticity on Population Differentiation.” Heredity 119, no. 4: 214–225. 28745716 10.1038/hdy.2017.36 PMC5597782

[ele70346-bib-0059] Schmid, M. , K. Tielbörger , A. Daval , and C. Mullon . 2026. “Data From: Plastic Germination, Temporal Niche Partitioning, and Emergent Assortative Mating in Annual Plants.” 10.6084/m9.figshare.30196897.41714578

[ele70346-bib-0060] Servedio, M. R. , G. S. V. Doorn , M. Kopp , A. M. Frame , and P. Nosil . 2011. “Magic Traits in Speciation: ‘Magic’ but Not Rare?” Trends in Ecology & Evolution 26, no. 8: 389–397. 21592615 10.1016/j.tree.2011.04.005

[ele70346-bib-0061] Simons, A. M. 2014. “Playing Smart vs. Playing Safe: The Joint Expression of Phenotypic Plasticity and Potential Bet Hedging Across and Within Thermal Environments.” Journal of Evolutionary Biology 27, no. 6: 1047–1056. 24739081 10.1111/jeb.12378

[ele70346-bib-0062] Sinervo, B. , and E. Svensson . 2002. “Correlational Selection and the Evolution of Genomic Architecture.” Heredity 89, no. 5: 329–338. 12399990 10.1038/sj.hdy.6800148

[ele70346-bib-0063] Slatkin, M. , and R. Lande . 1994. “Segregation Variance After Hybridization of Isolated Populations.” Genetical Research 64, no. 1: 51–56. 7958831 10.1017/s0016672300032547

[ele70346-bib-0064] Snyder, R. E. , and P. B. Adler . 2011. “Coexistence and Coevolution in Fluctuating Environments: Can the Storage Effect Evolve?” American Naturalist 178, no. 4: E76–E84. 10.1086/661905 21956035

[ele70346-bib-0065] Sommer, R. J. 2020. “Phenotypic Plasticity: From Theory and Genetics to Current and Future Challenges.” Genetics 215, no. 1: 1–13. 32371438 10.1534/genetics.120.303163 PMC7198268

[ele70346-bib-0066] Svardal, H. , C. Rueffler , and J. Hermisson . 2015. “A General Condition for Adaptive Genetic Polymorphism in Temporally and Spatially Heterogeneous Environments.” Theoretical Population Biology 99: 76–97. 25446960 10.1016/j.tpb.2014.11.002

[ele70346-bib-0067] Tielbörger, K. , M. C. Bilton , J. Metz , et al. 2014. “Middle‐Eastern Plant Communities Tolerate 9 Years of Drought in a Multi‐Site Climate Manipulation Experiment.” Nature Communications 5, no. 1: 5102. 10.1038/ncomms6102 PMC4205856 25283495

[ele70346-bib-0068] Tielbörger, K. , and M. Petrů . 2010. “An Experimental Test for Effects of the Maternal Environment on Delayed Germination.” Journal of Ecology 98, no. 5: 1216–1223.

[ele70346-bib-0069] Tielbörger, K. , and R. Prasse . 2009. “Do Seeds Sense Each Other? Testing for Density‐Dependent Germination in Desert Perennial Plants.” Oikos 118, no. 5: 792–800.

[ele70346-bib-0070] Tielbörger, K. , and A. Valleriani . 2005. “Can Seeds Predict Their Future? Germination Strategies of Density‐Regulated Desert Annuals.” Oikos 111, no. 2: 235–244.

[ele70346-bib-0071] Tufto, J. 2000. “The Evolution of Plasticity and Nonplastic Spatial and Temporal Adaptations in the Presence of Imperfect Environmental Cues.” American Naturalist 156, no. 2: 121–130. 10.1086/303381 10856196

[ele70346-bib-0072] Turcotte, M. M. , and J. M. Levine . 2016. “Phenotypic Plasticity and Species Coexistence.” Trends in Ecology & Evolution 31, no. 10: 803–813. 27527257 10.1016/j.tree.2016.07.013

[ele70346-bib-0073] Ummenhofer, C. C. , and G. A. Meehl . 2017. “Extreme Weather and Climate Events With Ecological Relevance: A Review.” Philosophical Transactions of the Royal Society, B: Biological Sciences 372, no. 1723: 20160135. 10.1098/rstb.2016.0135 PMC5434087 28483866

[ele70346-bib-0074] van Buskirk, J. , and U. K. Steiner . 2009. “The Fitness Costs of Developmental Canalization and Plasticity.” Journal of Evolutionary Biology 22, no. 4: 852–860. 19226418 10.1111/j.1420-9101.2009.01685.x

[ele70346-bib-0075] Venable, D. L. , and L. Lawlor . 1980. “Delayed Germination and Dispersal in Desert Annuals: Escape in Space and Time.” Oecologia 46, no. 2: 272–282. 28309684 10.1007/BF00540137

[ele70346-bib-0076] Venable, D. L. , C. E. Pake , and A. C. Caprio . 1993. “Diversity and Coexistence of Sonoran Desert Winter Annuals.” Plant Species Biology 8, no. 2–3: 207–216.

[ele70346-bib-0077] Via, S. , R. Gomulkiewicz , G. De Jong , S. M. Scheiner , C. D. Schlichting , and P. H. Van Tienderen . 1995. “Adaptive Phenotypic Plasticity: Consensus and Controversy.” Trends in Ecology & Evolution 10, no. 5: 212–217. 21237012 10.1016/s0169-5347(00)89061-8

[ele70346-bib-0078] Via, S. , and R. Lande . 1985. “Genotype‐Environment Interaction and the Evolution of Phenotypic Plasticity.” Evolution 39, no. 3: 505–522. 28561964 10.1111/j.1558-5646.1985.tb00391.x

[ele70346-bib-0079] Visser, M. E. , and P. Gienapp . 2019. “Evolutionary and Demographic Consequences of Phenological Mismatches.” Nature Ecology & Evolution 3: 879–885. 31011176 10.1038/s41559-019-0880-8 PMC6544530

[ele70346-bib-0080] West‐Eberhard, M. J. 1989. “Phenotypic Plasticity and the Origins of Diversity.” Annual Review of Ecology and Systematics 20, no. 1: 249–278.

[ele70346-bib-0081] Willis, C. G. , C. C. Baskin , J. M. Baskin , et al. 2014. “The Evolution of Seed Dormancy: Environmental Cues, Evolutionary Hubs, and Diversification of the Seed Plants.” New Phytologist 203, no. 1: 300–309. 24684268 10.1111/nph.12782

[ele70346-bib-0082] Wisnoski, N. I. , and L. G. Shoemaker . 2022. “Seed Banks Alter Metacommunity Diversity: The Interactive Effects of Competition, Dispersal and Dormancy.” Ecology Letters 25, no. 4: 740–753. 34965013 10.1111/ele.13944

[ele70346-bib-0083] Woltereck, R. 1909. “Weitere experimentelle Untersuchungen über Artveränderung, speziell über das Wesen quantitativer Artunterschiede bei Daphnien.” Verhandlungen der Deutschen Zoologischen Gesellschaft 19: 110–173.

[ele70346-bib-0084] Yamamichi, M. , and M. Hoso . 2017. “Roles of Maternal Effects in Maintaining Genetic Variation: Maternal Storage Effect.” Evolution 71, no. 2: 449–457. 27859045 10.1111/evo.13118

[ele70346-bib-0085] Yamamichi, M. , N. G. H. Jr , M. Rees , and S. P. Ellner . 2019. “Rapid Evolution With Generation Overlap: The Double‐Edged Effect of Dormancy.” Theoretical Ecology 12, no. 2: 179–195.

[ele70346-bib-0086] Yamamichi, M. , A. D. Letten , and S. J. Schreiber . 2023. “Eco‐Evolutionary Maintenance of Diversity in Fluctuating Environments.” Ecology Letters 26, no. S1: S152–S167. 37840028 10.1111/ele.14286

