# Supplementary material for: Plastic Germination, Temporal Niche Partitioning and Emergent Assortative Mating in Annual Plants
Source: Ecol Lett. 2026 Feb 19;29(2):e70346. doi: 10.1111/ele.70346 (PMC12920274; doi:10.1111/ele.70346)
Supplement: Supplementary file 1 — Data S1: ele70346‐sup‐0001‐supinfo.pdf. [file ELE-29-0-s001.pdf]

Plastic germination, temporal niche partitioning, and emergent assortative mating in annual plants

Max Schmid[1], Katja Tielbörger[1,*], Amaël Daval[1] and Charles Mullon[2]

[1]Institute of Evolution and Ecology, University of Tübingen, 72076 Tübingen, Germany
[2]Department of Ecology and Evolution, University of Lausanne, 1015 Lausanne, Switzerland

[*] Correspondence: katja.tielboerger@uni-tuebingen.de

A Supplementary Appendix - Environmental variation

A.1 Bivariate normal distribution

The environmental conditions during germination and reproduction $\boldsymbol{\theta}_t = (\theta_{g,t}, \theta_{f,t})$ for each year t are picked from a Bivariate normal distribution

$$\boldsymbol{\theta}_t \sim \mathcal{N}_2(\boldsymbol{\mu}, \boldsymbol{\Sigma}) \tag{A1}$$

with the vector of average environmental conditions

$$\boldsymbol{\mu} = \begin{pmatrix} \overline{\theta} \\ \overline{\theta} \end{pmatrix} \tag{A2}$$

and environmental variance-covariance matrix

$$\boldsymbol{\Sigma} = \begin{pmatrix} V & V_{gf} \\ V_{gf} & V \end{pmatrix} = \begin{pmatrix} V & \rho V \\ \rho V & V \end{pmatrix} . \tag{A3}$$

The environmental distribution thus is characterized by between-year environmental variance V (see eq. A7), the covariance between the germination and fecundity environment V_{gf} (see eq. A8), and the within-year correlation coefficient ρ. Note that the model is flexible with respect to the units and scales of $\theta_{g,t}$ and $\theta_{f,t}$, such that $\boldsymbol{\theta}_t$ might apply to a wide range of environmental parameters (e.g., temperature in K or °C, or precipitation amount of the first rain event or averaged over the course of a year).

With a bivariate normal distribution, the probability density function of $\theta_{g,t}$ and $\theta_{f,t}$ is

$$p(\boldsymbol{\theta}_t) = \frac{1}{2\pi\sqrt{1-\rho^2}V} \exp\left(-\frac{\left(\theta_{f,t}-\overline{\theta}\right)^2 - 2\rho\left(\theta_{f,t}-\overline{\theta}\right)\left(\theta_{g,t}-\overline{\theta}\right) + \left(\theta_{g,t}-\overline{\theta}\right)^2}{2\left(1-\rho^2\right)V}\right) , \tag{A4}$$

where $\theta_{\mathrm{g},t}$ and $\theta_{\mathrm{f},t}$ are correlated to each other with correlation coefficient ρ (e.g., eq. 46.1 in Kotz et al., 2000). When considering the long-term distribution of each environmental value separately, both $\theta_{\mathrm{g},t}$ and $\theta_{\mathrm{f},t}$ follow a univariate normal distribution with probability density function

$$\begin{aligned} p(\theta_{\mathrm{g},t}) &= \frac{1}{\sqrt{2\pi V}} \exp\left(-\frac{\left(\theta_{\mathrm{g},t} - \overline{\theta}\right)^2}{2V} \right) \\ p(\theta_{\mathrm{f},t}) &= \frac{1}{\sqrt{2\pi V}} \exp\left(-\frac{\left(\theta_{\mathrm{f},t} - \overline{\theta}\right)^2}{2V} \right) \end{aligned} . \tag{A5}$$

Both environmental conditions share the same average value $\overline{\theta}$ across years

$$\overline{\theta} = \mathrm{E}\left[\theta_{\mathrm{g},t}\right] = \mathrm{E}\left[\theta_{\mathrm{f},t}\right] , \tag{A6}$$

and the same between-year variance V

$$V = \mathrm{E}\left[\left(\theta_{\mathrm{g},t} - \overline{\theta}\right)^2\right] = \mathrm{E}\left[\left(\theta_{\mathrm{f},t} - \overline{\theta}\right)^2\right] , \tag{A7}$$

where expectations are taken over $p(\boldsymbol{\theta}_t)$ throughout. The covariance among the germination environment $\theta_{\mathrm{g},t}$ and the reproduction environment $\theta_{\mathrm{f},t}$ is

$$V_{\mathrm{gf}} = \mathrm{E}\left[\left(\theta_{\mathrm{g},t} - \overline{\theta}\right)\left(\theta_{\mathrm{f},t} - \overline{\theta}\right)\right] . \tag{A8}$$

A.2 Conditional distribution

To illustrate how environmental conditions change within each year, we here provide the conditional distribution of the fecundity environment $\theta_{\mathrm{f},t}$ given the germination environment $\theta_{\mathrm{g},t}$. This conditional distribution follows the univariate normal distribution

$$\theta_{\mathrm{f},t}\Big|_{\theta_{\mathrm{g},t}} \sim \mathcal{N}_1\left(\overline{\theta} + \rho\left(\theta_{\mathrm{g},t} - \overline{\theta}\right), \left(1 - \rho^2\right) V\right) . \tag{A9}$$

This makes it clear that the degree of environmental correlation ρ re-scales the conditional mean $\overline{\theta} + \rho\left(\theta_{\mathrm{g},t} - \overline{\theta}\right)$ and the conditional variance $\left(1 - \rho^2\right) V$. In the limiting case of $\rho \to 1$, the conditional distribution tends to

$$\theta_{\mathrm{f},t}\Big|_{\theta_{\mathrm{g},t},\, \rho=1} \sim \mathcal{N}_1\left(\theta_{\mathrm{g},t}, 0\right) , \tag{A10}$$

and in the case of $\rho \to -1$ to

$$\theta_{\mathrm{f},t}\Big|_{\theta_{\mathrm{g},t},\ \rho=-1} \sim \mathcal{N}_1\left(\overline{\theta} - \left(\theta_{\mathrm{g},t} - \overline{\theta}\right),\ 0\right) \ . \tag{A11}$$

These two cases illustrate that a large positive environmental correlation leads to constant environmental conditions within years (i.e. $\theta_{\mathrm{g},t} \to \theta_{\mathrm{f},t}$ with $\rho \to 1$), while large negative environmental correlation installs large within-year environmental changes (i.e. $\theta_{\mathrm{g},t} \to \overline{\theta} - \left(\theta_{\mathrm{g},t} - \overline{\theta}\right)$ with $\rho \to -1$).

B Supplementary Appendix - Single-trait case

B.1 Analysis

We first investigate the *single-trait case*, where germination and fecundity are controlled by the same trait ($z_{\mathrm{g},i} = z_{\mathrm{f},i} = z_i$). We study the evolution of this trait using an evolutionary invasion analysis. To this end, we consider a rare mutant with trait value z_{m} appearing in a large population that is otherwise monomorphic for a resident trait z (with mutational effect being small such that $|z_{\mathrm{m}} - z|$ is small). Whether or not the mutant can invade and substitute the resident trait value, can be determined from the mutants' geometric-mean growth rate, i.e.

$$W(z_{\mathrm{m}}, z) = \mathrm{E}\left[\log\left(w(z_{\mathrm{m}}, z, \boldsymbol{\theta}_t)\right)\right] \tag{B1}$$

(e.g., see Lewontin and Cohen, 1969; Sæther and Engen, 2015) where

$$w(z_{\mathrm{m}}, z, \boldsymbol{\theta}_t) \ = \ \gamma\left(z_{\mathrm{m}}, \theta_{\mathrm{g},t}\right) + \left(1 - \gamma\left(z, \theta_{\mathrm{g},t}\right)\right) \frac{g\left(z_{\mathrm{m}}, \theta_{\mathrm{g},t}\right) f\left(z_{\mathrm{m}}, \theta_{\mathrm{f},t}\right)}{g\left(z, \theta_{\mathrm{g},t}\right) f\left(z, \theta_{\mathrm{f},t}\right)} \ . \tag{B2}$$

is individual fitness, i.e. the expected number of seeds that a mutant seed in the seed bank at the beginning of year t contributes to next year's seed bank. Following the life cycle described in section "*Life cycle and traits*" of the main text, individual fitness (eq. B2) is composed of two parts reflecting two potential contributions. First, the mutant seed may stay in the seed bank (i.e. not germinate) and survive there to the next year, which occurs with probability

$$\gamma\left(z_{\mathrm{m}}, \theta_{\mathrm{g},t}\right) = \left(1 - g\left(z_{\mathrm{m}}, \theta_{\mathrm{g},t}\right)\right) s \tag{B3}$$

(where $g\left(z_{\mathrm{m}}, \theta_{\mathrm{g},t}\right)$ is given by eq. 1 in the main text). The second term of eq. (B2), meanwhile, captures that the mutant seed may germinate, reproduce with fecundity $f\left(z_{\mathrm{m}}, \theta_{\mathrm{f},t}\right)$ (given by eq. 2 in the main text), and contribute new seeds to the seed bank of the next year, competing with residents for $\left(1 - \gamma\left(z, \theta_{\mathrm{g},t}\right)\right)$ sites. See Chesson and Warner (1981) and Svardal et al. (2011) for similar formulations

of individual fitness.

Eq. (B2) assumes that the number of seeds in the seed bank N is constant across years. This assumption greatly simplifies the mathematical analysis but is biologically unrealistic, as seed-bank size will usually vary from year to year. Kortessis and Chesson (2021) addressed this by introducing competition among germinated plants for seed (or biomass) production (their eqs. 2–3). We follow a similar idea using simulations below and show that our main results still hold when seed-bank size is allowed to vary this way.

Invasion fitness $W(z_{\mathrm{m}}, z)$ can be used to infer on long-term evolution via a sequence of invasions of mutations (Geritz et al., 1998). The selection gradient

$$S(z) = \left.\frac{\partial W(z_{\mathrm{m}}, z)}{\partial z_{\mathrm{m}}}\right|_{z_{\mathrm{m}}=z} \tag{B4}$$

indicates directional selection during this sequence, with selection favoring trait increase when $S(z) > 0$ and trait decrease when $S(z) < 0$. A strategy z^* where $S(z^*) = 0$ is called a singular strategy. Whether or not such a singular strategy will be approached by gradual evolution is determined by the sign of

$$C(z^*) = \left.\frac{dS(z)}{dz}\right|_{z=z^*}, \tag{B5}$$

with $C(z^*) < 0$ indicating that z^* is an attractor (i.e. is convergence stable) and $C(z^*) > 0$ indicating that z^* is a repellor of evolutionary dynamics. Once a population has converged to an attractor z^*, the disruptive selection coefficient

$$H(z^*) = \left.\frac{\partial^2 W(z_{\mathrm{m}}, z)}{\partial z_{\mathrm{m}}^2}\right|_{z_{\mathrm{m}}=z=z^*} \tag{B6}$$

determines whether the population remains monomorphic (when $H(z^*) < 0$) or becomes polymorphic in a process referred to as evolutionary branching (when $H(z^*) > 0$).

B.2 Results

B.2.1 Selection gradient

Substituting eq. (B1) into eq. (B4), we obtain the selection gradient

$$\begin{aligned} S(z) &= \mathrm{E}\left[\frac{\partial}{\partial z_{\mathrm{m}}}\log\left(\gamma\left(z_{\mathrm{m}}, \theta_{\mathrm{g},t}\right) + \left(1-\gamma\left(z, \theta_{\mathrm{g},t}\right)\right)\frac{g\left(z_{\mathrm{m}}, \theta_{\mathrm{g},t}\right) f\left(z_{\mathrm{m}}, \theta_{\mathrm{f},t}\right)}{g\left(z, \theta_{\mathrm{g},t}\right) f\left(z, \theta_{\mathrm{f},t}\right)}\right)\right]\Bigg|_{z_{\mathrm{m}}=z} \\ &= \mathrm{E}\left[(1-s)\left(\frac{1}{g\left(z, \theta_{\mathrm{g},t}\right)}\frac{\partial g\left(z_{\mathrm{m}}, \theta_{\mathrm{g},t}\right)}{\partial z_{\mathrm{m}}}\right) + \left(1-\gamma\left(z, \theta_{\mathrm{g},t}\right)\right)\left(\frac{1}{f\left(z, \theta_{\mathrm{f},t}\right)}\frac{\partial f\left(z_{\mathrm{m}}, \theta_{\mathrm{f},t}\right)}{\partial z_{\mathrm{m}}}\right)\right]\Bigg|_{z_{\mathrm{m}}=z} \end{aligned} \tag{B7}$$

with the derivatives being

$$\begin{aligned}\frac{1}{g\left(z,\theta_{\mathrm{g},t}\right)}\frac{\partial g\left(z_{\mathrm{m}},\theta_{\mathrm{g},t}\right)}{\partial z_{\mathrm{m}}}\bigg|_{z_{\mathrm{m}}=z} &= -\frac{z-\theta_{\mathrm{g},t}}{\sigma_{\mathrm{g}}^2}\\ \frac{1}{f\left(z,\theta_{\mathrm{f},t}\right)}\frac{\partial f\left(z_{\mathrm{m}},\theta_{\mathrm{f},t}\right)}{\partial z_{\mathrm{m}}}\bigg|_{z_{\mathrm{m}}=z} &= -\frac{z-\theta_{\mathrm{f},t}}{\sigma_{\mathrm{f}}^2}\end{aligned} \quad . \tag{B8}$$

To take the expectation in eq. (B7), we substitute $1-\gamma\left(z,\theta_{\mathrm{g},t}\right) = 1-\left(1-g\left(z,\theta_{\mathrm{g},t}\right)\right)s$ (eq. B3) and compute the long-term average germination probability :

$$\mathrm{E}\left[g\left(z,\theta_{\mathrm{g},t}\right)\right] = \frac{g_{\max}}{\sqrt{\left(1+\dfrac{V}{\sigma_{\mathrm{g}}^2}\right)}}\,\mathrm{Exp}\left[-\frac{\left(z-\overline{\theta}\right)^2}{2\left(\sigma_{\mathrm{g}}^2+V\right)}\right] \quad , \tag{B9}$$

and the average product of germination probability and the environment during adult fecundity :

$$\mathrm{E}\left[g\left(z,\theta_{\mathrm{g},t}\right)\theta_{\mathrm{f},t}\right] = \mathrm{E}\left[g\left(z,\theta_{\mathrm{g},t}\right)\right]\left(\overline{\theta} + \rho\,\frac{V\left(z-\overline{\theta}\right)}{\left(V+\sigma_{\mathrm{g}}^2\right)}\right) \quad . \tag{B10}$$

Using eqs. (B8)-(B10) for eq. (B7), we obtain that the selection gradient can be written as

$$S(z) = -\frac{z-\overline{\theta}}{V+\sigma_{\mathrm{g}}^2}\left((1-s)\left(V+\sigma_{\mathrm{g}}^2\right)\left(\frac{1}{\sigma_{\mathrm{g}}^2}+\frac{1}{\sigma_{\mathrm{f}}^2}\right) + \mathrm{E}\left[g\left(z,\theta_{\mathrm{g},t}\right)\right]s\left(\frac{\left(1-\rho\right)V+\sigma_{\mathrm{g}}^2}{\sigma_{\mathrm{f}}^2}\right)\right) \quad , \tag{B11}$$

which is zero when $z = z^* = \overline{\theta}$. This singular strategy is convergence stable since

$$C(z^*) = -\left(\frac{g_{\max}\,s\,\sigma_{\mathrm{g}}^2}{\sigma_{\mathrm{f}}^2\left(V+\sigma_{\mathrm{g}}^2\right)^2}\left(\left(V(1-\rho)+\sigma_{\mathrm{g}}^2\right)\sqrt{\frac{V+\sigma_{\mathrm{g}}^2}{\sigma_{\mathrm{g}}^2}}\right)+(1-s)\left(\frac{1}{\sigma_{\mathrm{g}}^2}+\frac{1}{\sigma_{\mathrm{f}}^2}\right)\right) < 0, \tag{B12}$$

always. Therefore, the population will gradually evolve under directional selection to germinate under and be adapted to average conditions $\overline{\theta}$.

B.2.2 Disruptive selection coefficient

To determine whether the population will subsequently become polymorphic, we plug eq. (B1) into the eq. (B6) to obtain

$$\begin{aligned}H(z^*) &= \mathrm{E}\left[\frac{\partial^2}{\partial z_{\mathrm{m}}^2}\log\left(\gamma\left(z_{\mathrm{m}},\theta_{\mathrm{g},t}\right)+\left(1-\gamma\left(z,\theta_{\mathrm{g},t}\right)\right)\frac{g\left(z_{\mathrm{m}},\theta_{\mathrm{g},t}\right)f\left(z_{\mathrm{m}},\theta_{\mathrm{f},t}\right)}{g\left(z,\theta_{\mathrm{g},t}\right)f\left(z,\theta_{\mathrm{f},t}\right)}\right)\right]\Bigg|_{z_{\mathrm{m}}=z=z^*}\\ &= \mathrm{E}\Big[H_1+H_2+H_3\Big]\Big|_{z_{\mathrm{m}}=z=z^*}\end{aligned} \tag{B13}$$

where we have decomposed disruptive selection into the expectation of three effects

$$\begin{aligned}
H_1 &= \left(1-\gamma\left(z,\theta_{\mathrm{g},t}\right)\right)\left(\frac{1}{f\left(z,\theta_{\mathrm{f},t}\right)}\frac{\partial^2 f\left(z_{\mathrm{m}},\theta_{\mathrm{f},t}\right)}{\partial z_{\mathrm{m}}^2}\right)-\left(1-\gamma\left(z,\theta_{\mathrm{g},t}\right)\right)^2\left(\frac{1}{f\left(z,\theta_{\mathrm{f},t}\right)}\frac{\partial f\left(z_{\mathrm{m}},\theta_{\mathrm{f},t}\right)}{\partial z_{\mathrm{m}}}\right)^2\Bigg|_{z_{\mathrm{m}}=z}\\
&= \frac{\left(1-\gamma\left(z,\theta_{\mathrm{g},t}\right)\right)}{\sigma_{\mathrm{g}}^4}\left(\gamma\left(z,\theta_{\mathrm{g},t}\right)\left(z-\theta_{\mathrm{f},t}\right)^2-\sigma_{\mathrm{f}}^2\right)\\
H_2 &= (1-s)\left(\frac{1}{g\left(z,\theta_{\mathrm{g},t}\right)}\frac{\partial^2 g\left(z_{\mathrm{m}},\theta_{\mathrm{g},t}\right)}{\partial z_{\mathrm{m}}^2}\right)-(1-s)^2\left(\frac{1}{g\left(z,\theta_{\mathrm{g},t}\right)}\frac{\partial g\left(z_{\mathrm{m}},\theta_{\mathrm{g},t}\right)}{\partial z_{\mathrm{m}}}\right)^2\Bigg|_{z_{\mathrm{m}}=z}\\
&= \frac{(1-s)}{\sigma_{\mathrm{g}}^4}\left(s\left(z-\theta_{\mathrm{g},t}\right)^2-\sigma_{\mathrm{g}}^2\right)\\
H_3 &= 2\,s\left(1-\gamma\left(z,\theta_{\mathrm{g},t}\right)\right)\left(\frac{1}{f\left(z,\theta_{\mathrm{f},t}\right)}\frac{\partial f\left(z_{\mathrm{m}},\theta_{\mathrm{f},t}\right)}{\partial z_{\mathrm{m}}}\frac{1}{g\left(z,\theta_{\mathrm{g},t}\right)}\frac{\partial g\left(z_{\mathrm{m}},\theta_{\mathrm{g},t}\right)}{\partial z_{\mathrm{m}}}\right)\Bigg|_{z_{\mathrm{m}}=z}\\
&= 2\,s\,\frac{\left(1-\gamma\left(z,\theta_{\mathrm{g},t}\right)\right)}{\sigma_{\mathrm{g}}^2\,\sigma_{\mathrm{f}}^2}\left(z-\theta_{\mathrm{g},t}\right)\left(z-\theta_{\mathrm{f},t}\right).
\end{aligned} \tag{B14}$$

The first $\mathrm{E}[H_1]$ corresponds to disruptive selection on the trait due to its effect on fecundity, the second $\mathrm{E}[H_2]$ to its effect on germination, and the third $\mathrm{E}[H_3]$ due its interaction on fecundity and germination.

The averages of the different terms that appear in H_1, H_2, and H_3 can be written as

$$\begin{aligned}
\mathrm{E}\left[g\left(z,\theta_{\mathrm{g},t}\right)\right]\Big|_{z_{\mathrm{m}}=z=z^*} &= \frac{g_{\max}}{\sqrt{1+\dfrac{V}{\sigma_{\mathrm{g}}^2}}} \equiv \overline{g}\\
\mathrm{E}\left[g\left(z,\theta_{\mathrm{g},t}\right)^2\right]\Big|_{z_{\mathrm{m}}=z=z^*} &= \frac{g_{\max}^2}{\sqrt{1+\dfrac{2\,V}{\sigma_{\mathrm{g}}^2}}} \equiv \overline{g^2}\\
\mathrm{E}\left[g\left(z,\theta_{\mathrm{g},t}\right)\,\theta_{\mathrm{g},t}\right]\Big|_{z_{\mathrm{m}}=z=z^*} &= \overline{g}\,\overline{\theta}\\
\mathrm{E}\left[g\left(z,\theta_{\mathrm{g},t}\right)\,\theta_{\mathrm{f},t}\right]\Big|_{z_{\mathrm{m}}=z=z^*} &= \overline{g}\,\overline{\theta}\\
\mathrm{E}\left[g\left(z,\theta_{\mathrm{g},t}\right)^2\,\theta_{\mathrm{f},t}\right]\Big|_{z_{\mathrm{m}}=z=z^*} &= \overline{g^2}\,\overline{\theta}\\
\mathrm{E}\left[g\left(z,\theta_{\mathrm{g},t}\right)\,\theta_{\mathrm{f},t}^2\right]\Big|_{z_{\mathrm{m}}=z=z^*} &= \overline{g}\left(\overline{\theta}^2+V\,\frac{V\left(1-\rho^2\right)+\sigma_{\mathrm{g}}^2}{\left(V+\sigma_{\mathrm{g}}^2\right)}\right)\\
\mathrm{E}\left[g\left(z,\theta_{\mathrm{g},t}\right)^2\,\theta_{\mathrm{f},t}^2\right]\Big|_{z_{\mathrm{m}}=z=z^*} &= \overline{g^2}\left(\overline{\theta}^2+V\,\frac{2V\left(1-\rho^2\right)+\sigma_{\mathrm{g}}^2}{\left(2V+\sigma_{\mathrm{g}}^2\right)}\right)\\
\mathrm{E}\left[g\left(z,\theta_{\mathrm{g},t}\right)\,\theta_{\mathrm{g},t}\,\theta_{\mathrm{f},t}\right]\Big|_{z_{\mathrm{m}}=z=z^*} &= \overline{g}\left(\overline{\theta}^2+\rho\,\frac{V\,\sigma_{\mathrm{g}}^2}{V+\sigma_{\mathrm{g}}^2}\right)\\
\mathrm{E}\left[\theta_{\mathrm{g},t}\,\theta_{\mathrm{f},t}\right]\Big|_{z_{\mathrm{m}}=z=z^*} &= \overline{\theta}^2+\rho\,V
\end{aligned}, \tag{B15}$$

where we have used the overline as a shorthand for expectation over $p(\boldsymbol{\theta}_t)$ when $z=z^*$. Using these

expressions, the three components of $H(z^*)$ then read as

$$\begin{aligned}
\mathrm{E}[H_1] &= V\frac{s}{\sigma_\mathrm{f}^4}\left((1-s)-\overline{g^2}\,s\frac{2V\left(1-\rho^2\right)+\sigma_\mathrm{g}^2}{2V+\sigma_\mathrm{g}^2}-\overline{g}\left((1-2s)\,\frac{V\left(1-\rho^2\right)+\sigma_\mathrm{g}^2}{V+\sigma_\mathrm{g}^2}\right)\right)-\frac{\left(1-\overline{\gamma}\right)}{\sigma_\mathrm{f}^2}\\
\mathrm{E}[H_2] &= \frac{(1-s)}{\sigma_\mathrm{g}^4}\left(s\,V-\sigma_\mathrm{g}^2\right) \\
\mathrm{E}[H_3] &= 2\rho\,s\,V\,\frac{(1-s)V+\left(1-(1-\overline{g})s\right)\sigma_\mathrm{g}^2}{\sigma_\mathrm{f}^2\sigma_\mathrm{g}^2(V+\sigma_\mathrm{g}^2)}
\end{aligned}\quad, \tag{B16}$$

where we defined

$$\overline{\gamma}\;\equiv\;(1-\overline{g})\;s \tag{B17}$$

as the expected probability of staying and surviving in the seed bank.

Eq. (B16) is fairly complicated. Let us first consider the case without plastic germination ($\sigma_\mathrm{g}^2\to\infty$). The sum of the components in eq. (B16) then reduces to

$$H(z^*)\;=\;\frac{(1-(1-g_\mathrm{max})s)}{\sigma_\mathrm{f}^4}\left(\left(1-g_\mathrm{max}\right)\,s\,V-\sigma_\mathrm{f}^2\right)\;, \tag{B18}$$

where $(1-g_\mathrm{max})s$ is the generation overlap in this case. This recovers the classical result that polymorphism emerges when $(1-g_\mathrm{max})sV>\sigma_\mathrm{f}^2$ (Chesson, 1983; Ellner and Hairston, 1994; Svardal et al., 2015).

We now turn to the case with plasticity ($\sigma_\mathrm{g}^2<\infty$) but no environmental correlation within years ($\rho=0$). In this case, $\mathrm{E}[H_3]=0$ and the disruptive selection coefficient simplifies to

$$H(z^*)\;=\;\frac{1}{\sigma_\mathrm{f}^2}\left(\left(\left(1-\overline{\gamma}\right)-\overline{\left(1-\gamma\right)^2}\right)\frac{V}{\sigma_\mathrm{f}^2}-\left(1-\overline{\gamma}\right)\right)\;+\;\frac{1-s}{\sigma_\mathrm{g}^2}\left(s\,\frac{V}{\sigma_\mathrm{g}^2}-1\right)\;, \tag{B19}$$

where we defined

$$\overline{\left(1-\gamma\right)^2}\;\equiv\;\mathrm{E}\left[\left(1-(1-g(z^*,\theta_{\mathrm{g},t})s)\right)^2\right]\;=\;(1-s)^2\;+\;2\,\overline{g}\,s(1-s)\;+\;\overline{g^2}\,s^2\;. \tag{B20}$$

According to eq. (B19), plastic seed germination ($\sigma_\mathrm{g}^2<\infty$) at $\rho=0$ influences evolutionary branching through two components. The right-hand term shows that plasticity can either favour or oppose polymorphism depending on whether sV/σ_g^2 exceeds 1, that is, on the strength of between-year variance relative to the steepness of the germination reaction norm. When between-year variance is large ($sV>\sigma_\mathrm{g}^2$), greater plasticity accentuates differences in the years in which trait types are expressed, increasing $H(z^*)$ and favouring polymorphism. The left-hand term reflects how plasticity alters the contribution of adult fecundity to disruptive selection by changing the long-term germination prob-

abilities $\overline{g}$ and $\overline{g^2}$, and therefore $1-\overline{\gamma}$ and $\overline{(1-\gamma)^2}$. As $\overline{g}$ and $\overline{g^2}$ decrease with stronger plasticity, seeds spend more time in the seed bank and germinate in fewer years, altering their effective exposure to between-year variation in fecundity. The sign of this contribution depends on the balance between V and σ_{f}^2, so plasticity may either increase or decrease $H(z^*)$ through this component. Overall, plastic germination has no systematic effect on polymorphism when $\rho = 0$ because the environment at germination contains no information about conditions later in the year. Plasticity influences diversification only by altering how sensitively germination responds to between-year fluctuations and how long seeds remain dormant. Depending on parameter values, these effects can either strengthen or weaken disruptive selection, consistent with Fig. 2.

When germination and reproduction environments are correlated within years ($\rho \neq 0$), an additional effect enters through $\mathrm{E}[H_3]$ in eq. (B16). From eq. (B16), $\mathrm{E}[H_3]$ is proportional to ρ and to a strictly positive fraction, so its sign is the sign of ρ, and its magnitude increases with stronger plasticity (smaller σ_{g}^2). When $\rho > 0$, early- and late-season environments tend to favour the same trait values, and greater plasticity causes trait types to germinate predominantly in years that also maximise their fecundity. This increases $H(z^*)$ and promotes polymorphism. When $\rho < 0$, early- and late-season optima tend to be different each other, so plasticity causes specialised types to germinate in years that are unfavourable for their later reproduction, which reduces $H(z^*)$ and opposes polymorphism. See main text for more detailed explorations of the parameter space and explanations.

C Supplementary Appendix - Two-trait case

C.1 Analysis

In the *two-trait case*, we assume that the germination probability $g(z_{\mathrm{g},i}, \theta_{\mathrm{g},t})$ and adult fecundity $f(z_{\mathrm{f},i}, \theta_{\mathrm{f},t})$ of an individual are controlled by two separate traits, denoted by $z_{\mathrm{g},i}$ and $z_{\mathrm{f},i}$. These traits are controlled by two distinct loci, and mutations have independent effects on both traits (i.e. mutations only hit one trait at a time). We characterize the resident population by trait vector

$$\mathbf{z} = \begin{pmatrix} z_{\mathrm{g}} \\ z_{\mathrm{f}} \end{pmatrix} \tag{C1}$$

and the mutant individuals by trait vector

$$\mathbf{z_m} = \begin{pmatrix} z_{\mathrm{g,m}} \\ z_{\mathrm{f,m}} \end{pmatrix} . \tag{C2}$$

The mutants' geometric-mean growth rate in the *two-trait case* is

$$W(\mathbf{z_m},\mathbf{z}) = \mathrm{E}\left[\log\left(\gamma\left(z_{\mathrm{g,m}},\theta_{\mathrm{g},t}\right)+\left(1-\gamma\left(z_{\mathrm{g}},\theta_{\mathrm{g},t}\right)\right)\ \frac{g\left(z_{\mathrm{g,m}},\theta_{\mathrm{g},t}\right)f\left(z_{\mathrm{f,m}},\theta_{\mathrm{f},t}\right)}{g\left(z_{\mathrm{g}},\theta_{\mathrm{g},t}\right)f\left(z_{\mathrm{f}},\theta_{\mathrm{f},t}\right)}\right)\right] \quad . \tag{C3}$$

This expression is essentially the same as in eqs. (B1) and eq. (B2) but with two distinct traits control seed germination and fecundity, respectively.

Directional selection on trait z_{g} and z_{f} is characterized by the vector

$$\mathbf{S}(\mathbf{z}) = \begin{pmatrix} S_{\mathrm{g}}(\mathbf{z}) \\ S_{\mathrm{f}}(\mathbf{z}) \end{pmatrix} = \begin{pmatrix} \left.\frac{\partial W(\mathbf{z_m},\mathbf{z})}{\partial z_{\mathrm{g,m}}}\right|_{\mathbf{z_m}=\mathbf{z}} \\ \left.\frac{\partial W(\mathbf{z_m},\mathbf{z})}{\partial z_{\mathrm{f,m}}}\right|_{\mathbf{z_m}=\mathbf{z}} \end{pmatrix} , \tag{C4}$$

which points in the direction that selection favors in two-trait space. Directional selection thus vanishes at a singular strategy $\mathbf{z}^* = (z_{\mathrm{g}}^*, z_{\mathrm{f}}^*)$ such that $S_{\mathrm{g}}(\mathbf{z}^*) = 0$ and $S_{\mathrm{f}}(\mathbf{z}^*) = 0$. Assuming that both traits mutate independently, a singular strategy is an attractor of directional selection when all eigenvalues of the Jacobian matrix

$$\mathbf{J}\left(\mathbf{z}^*\right) = \begin{pmatrix} \left.\frac{\partial S_{\mathrm{g}}(\mathbf{z})}{\partial z_{\mathrm{g}}}\right|_{\mathbf{z}=\mathbf{z}^*} & \left.\frac{\partial S_{\mathrm{g}}(\mathbf{z})}{\partial z_{\mathrm{f}}}\right|_{\mathbf{z}=\mathbf{z}^*} \\ \left.\frac{\partial S_{\mathrm{f}}(\mathbf{z})}{\partial z_{\mathrm{g}}}\right|_{\mathbf{z}=\mathbf{z}^*} & \left.\frac{\partial S_{\mathrm{f}}(\mathbf{z})}{\partial z_{\mathrm{f}}}\right|_{\mathbf{z}=\mathbf{z}^*} \end{pmatrix} \tag{C5}$$

have negative real parts, in which case $\mathbf{z}^*$ is convergence stable (Leimar, 2009). Once the population expresses $\mathbf{z}^*$, the population experiences disruptive selection and may thus become polymorphic when the leading eigenvalue $\lambda(\mathbf{z}^*)$ of the Hessian matrix

$$\mathbf{H}\left(\mathbf{z}^*\right) = \begin{pmatrix} H_{\mathrm{gg}}\left(\mathbf{z}^*\right) & H_{\mathrm{gf}}\left(\mathbf{z}^*\right) \\ H_{\mathrm{fg}}\left(\mathbf{z}^*\right) & H_{\mathrm{ff}}\left(\mathbf{z}^*\right) \end{pmatrix} = \begin{pmatrix} \left.\frac{\partial^2 W(\mathbf{z_m},\mathbf{z})}{\partial z_{\mathrm{g,m}}^2}\right|_{\mathbf{z_m}=\mathbf{z}^*} & \left.\frac{\partial^2 W(\mathbf{z_m},\mathbf{z})}{\partial z_{\mathrm{f,m}}\partial z_{\mathrm{g,m}}}\right|_{\mathbf{z_m}=\mathbf{z}^*} \\ \left.\frac{\partial^2 W(\mathbf{z_m},\mathbf{z})}{\partial z_{\mathrm{g,m}}\partial z_{\mathrm{f,m}}}\right|_{\mathbf{z_m}=\mathbf{z}^*} & \left.\frac{\partial^2 W(\mathbf{z_m},\mathbf{z})}{\partial z_{\mathrm{f,m}}^2}\right|_{\mathbf{z_m}=\mathbf{z}^*} \end{pmatrix} \tag{C6}$$

is positive ($\lambda(\mathbf{z}^*) > 0$). Otherwise, the population remains monomorphic under stabilizing selection when $\lambda(\mathbf{z}^*) < 0$.

C.2 Results

C.2.1 Selection gradient

Following eqs. (C3) and (C4), the selection gradient in the two-trait model becomes

$$\mathbf{S}(\mathbf{z}) = \mathrm{E}\left[\left.\left(\begin{array}{c} (1-s)\,\dfrac{1}{g\left(z_{\mathrm{g}},\theta_{\mathrm{g},t}\right)}\dfrac{\partial g\left(z_{\mathrm{g,m}},\theta_{\mathrm{g},t}\right)}{\partial z_{\mathrm{g,m}}} \\ \left(1-\gamma\left(z_{\mathrm{g}},\theta_{\mathrm{g},t}\right)\right)\dfrac{1}{f\left(z_{\mathrm{f}},\theta_{\mathrm{f},t}\right)}\dfrac{\partial f\left(z_{\mathrm{f,m}},\theta_{\mathrm{f},t}\right)}{\partial z_{\mathrm{f,m}}}\end{array}\right)\right]\right|_{\mathbf{z_m}=\mathbf{z}}, \tag{C7}$$

with the derivatives

$$\begin{aligned} \frac{1}{g\left(z_{\mathrm{g}},\theta_{\mathrm{g},t}\right)}\frac{\partial g\left(z_{\mathrm{g,m}},\theta_{\mathrm{g},t}\right)}{\partial z_{\mathrm{g,m}}}\bigg|_{\mathbf{z_m}=\mathbf{z}} &= -\frac{\left(z_{\mathrm{g}}-\theta_{\mathrm{g},t}\right)}{\sigma_{\mathrm{g}}^2} \\ \frac{1}{f\left(z_{\mathrm{f}},\theta_{\mathrm{f},t}\right)}\frac{\partial f\left(z_{\mathrm{f,m}},\theta_{\mathrm{f},t}\right)}{\partial z_{\mathrm{f,m}}}\bigg|_{\mathbf{z_m}=\mathbf{z}} &= -\frac{\left(z_{\mathrm{f}}-\theta_{\mathrm{f},t}\right)}{\sigma_{\mathrm{f}}^2} \end{aligned}. \tag{C8}$$

For a population that is monomorphic for the trait vector **z**, the long-term average germination probability and the long-term average product of germination probability and fecundity environment is

$$\begin{aligned} \mathrm{E}\left[g\left(z_{\mathrm{g}},\theta_{\mathrm{g},t}\right)\right] &= \frac{g_{\max}}{\sqrt{\left(1+\dfrac{V}{\sigma_{\mathrm{g}}^2}\right)}}\,\mathrm{Exp}\left[-\frac{\left(z_{\mathrm{g}}-\overline{\theta}\right)^2}{2\left(\sigma_{\mathrm{g}}^2+V\right)}\right] \\ \mathrm{E}\left[g\left(z_{\mathrm{g}},\theta_{\mathrm{g},t}\right)\,\theta_{\mathrm{f},t}\right] &= \mathrm{E}\left[g\left(z_{\mathrm{g}},\theta_{\mathrm{g},t}\right)\right]\left(\overline{\theta}+\rho\,\frac{V\left(z_{\mathrm{g}}-\overline{\theta}\right)}{\left(\sigma_{\mathrm{g}}^2+V\right)}\right) \end{aligned}, \tag{C9}$$

with the expectations being taken over $p(\boldsymbol{\theta}_t)$, respectively. The selection gradient then becomes

$$\mathbf{S}(\mathbf{z}) = \left(\begin{array}{c} -\left(z_{\mathrm{g}}-\overline{\theta}\right)\dfrac{(1-s)}{\sigma_{\mathrm{g}}^2} \\ -\left(z_{\mathrm{f}}-\overline{\theta}\right)\dfrac{\left(1-\left(1-\mathrm{E}\left[g\left(z_{\mathrm{g}},\theta_{\mathrm{g},t}\right)\right]\right)s\right)}{\sigma_{\mathrm{f}}^2}+\rho\left(z_{\mathrm{g}}-\overline{\theta}\right)\dfrac{\mathrm{E}\left[g\left(z_{\mathrm{g}},\theta_{\mathrm{g},t}\right)\right]}{\sigma_{\mathrm{f}}^2\left(V+\sigma_{\mathrm{g}}^2\right)}sV\end{array}\right), \tag{C10}$$

with the singular strategy

$$\mathbf{z}^* = \begin{pmatrix} z_{\mathrm{g}}^* \\ z_{\mathrm{f}}^* \end{pmatrix} = \begin{pmatrix} \overline{\theta} \\ \overline{\theta} \end{pmatrix}. \tag{C11}$$

This singular strategy is convergence stable because the eigenvalues of the Jacobian

$$\mathbf{J}\left(\mathbf{z}^*\right) = \begin{pmatrix} -\dfrac{(1-s)}{\sigma_{\mathrm{g}}^2} & 0 \\ \rho\,\overline{g}\,s\,\dfrac{V}{\sigma_{\mathrm{f}}^2\left(\sigma_{\mathrm{g}}^2+V\right)} & -\dfrac{\left(1-\left(1-\overline{g}\right)s\right)}{\sigma_{\mathrm{f}}^2} \end{pmatrix} \tag{C12}$$

are both negative (with eigenvalues equal to $-(1-s)/\sigma_{\mathrm{g}}^2$ and $-\left(1-\left(1-\overline{g}\right)s\right)/\sigma_{\mathrm{f}}^2$, where $\overline{g}$ is given by eq. C15).

C.2.2 Disruptive selection coefficient

The entries of the Hessian matrix, following expressions (C3) and (C6), become

$$\begin{aligned}
H_{\mathrm{gg}}\left(\mathbf{z}^*\right) &= \mathrm{E}\left[(1-s)\left(\frac{1}{g\left(z_{\mathrm{g}},\theta_{\mathrm{g},t}\right)}\frac{\partial^2 g\left(z_{\mathrm{g,m}},\theta_{\mathrm{g},t}\right)}{\partial z_{\mathrm{g,m}}^2}-(1-s)\left(\frac{1}{g\left(z_{\mathrm{g}},\theta_{\mathrm{g},t}\right)}\frac{\partial g\left(z_{\mathrm{g,m}},\theta_{\mathrm{g},t}\right)}{\partial z_{\mathrm{g,m}}}\right)^2\right)\right]\Bigg|_{\mathbf{z_m}=\mathbf{z}=\mathbf{z}^*} \\
H_{\mathrm{ff}}\left(\mathbf{z}^*\right) &= \mathrm{E}\left[\left(1-\gamma\left(z_{\mathrm{g}},\theta_{\mathrm{g},t}\right)\right)\left(\frac{1}{f\left(z_{\mathrm{f}},\theta_{\mathrm{f},t}\right)}\frac{\partial^2 f\left(z_{\mathrm{f,m}},\theta_{\mathrm{f},t}\right)}{\partial z_{\mathrm{f,m}}^2}-\left(1-\gamma\left(z_{\mathrm{g}},\theta_{\mathrm{g},t}\right)\right)\left(\frac{1}{f\left(z_{\mathrm{f}},\theta_{\mathrm{f},t}\right)}\frac{\partial f\left(z_{\mathrm{f,m}},\theta_{\mathrm{f},t}\right)}{\partial z_{\mathrm{f,m}}}\right)^2\right)\right]\Bigg|_{\mathbf{z_m}=\mathbf{z}=\mathbf{z}^*} \\
H_{\mathrm{gf}}\left(\mathbf{z}^*\right) &= \mathrm{E}\left[s\left(1-\gamma\left(z_{\mathrm{g}},\theta_{\mathrm{g},t}\right)\right)\left(\frac{1}{g\left(z_{\mathrm{g}},\theta_{\mathrm{g},t}\right)}\frac{\partial g\left(z_{\mathrm{g,m}},\theta_{\mathrm{g},t}\right)}{\partial z_{\mathrm{g,m}}}\frac{1}{f\left(z_{\mathrm{f}},\theta_{\mathrm{f},t}\right)}\frac{\partial f\left(z_{\mathrm{f,m}},\theta_{\mathrm{f},t}\right)}{\partial z_{\mathrm{f,m}}}\right)\right]\Bigg|_{\mathbf{z_m}=\mathbf{z}=\mathbf{z}^*}
\end{aligned} \tag{C13}$$

with the first order derivatives following eqs. (C8) and the second order derivatives being

$$\begin{aligned}
\frac{1}{g\left(z_{\mathrm{g}},\theta_{\mathrm{g},t}\right)}\frac{\partial^2 g\left(z_{\mathrm{g,m}},\theta_{\mathrm{g},t}\right)}{\partial z_{\mathrm{g,m}}^2}\Bigg|_{\mathbf{z_m}=\mathbf{z}} &= \frac{\left(z_{\mathrm{g}}-\theta_{\mathrm{g},t}\right)^2-\sigma_{\mathrm{g}}^2}{\sigma_{\mathrm{g}}^4} \\
\frac{1}{f\left(z_{\mathrm{f}},\theta_{\mathrm{f},t}\right)}\frac{\partial^2 f\left(z_{\mathrm{f,m}},\theta_{\mathrm{f},t}\right)}{\partial z_{\mathrm{f,m}}^2}\Bigg|_{\mathbf{z_m}=\mathbf{z}} &= \frac{\left(z_{\mathrm{f}}-\theta_{\mathrm{f},t}\right)^2-\sigma_{\mathrm{f}}^2}{\sigma_{\mathrm{f}}^4}
\end{aligned}. \tag{C14}$$

The relevant terms appearing in the Hessian matrix, once averaged over environments, can be expressed as

$$
\begin{aligned}
\mathrm{E}\left[g\left(z_{\mathrm{g}},\theta_{\mathrm{g},t}\right)\right]\Big|_{\mathbf{z_m}=\mathbf{z}=\mathbf{z}^*} &= \frac{g_{\max}}{\sqrt{1+\dfrac{V}{\sigma_{\mathrm{g}}^2}}} \equiv \overline{g} \\
\mathrm{E}\left[g\left(z_{\mathrm{g}},\theta_{\mathrm{g},t}\right)^2\right]\Big|_{\mathbf{z_m}=\mathbf{z}=\mathbf{z}^*} &= \frac{g_{\max}^2}{\sqrt{1+\dfrac{2\,V}{\sigma_{\mathrm{g}}^2}}} \equiv \overline{g^2} \\
\mathrm{E}\left[g\left(z_{\mathrm{g}},\theta_{\mathrm{g},t}\right)\,\theta_{\mathrm{g},t}\right]\Big|_{\mathbf{z_m}=\mathbf{z}=\mathbf{z}^*} &= \overline{g}\,\overline{\theta} \qquad\qquad , \\
\mathrm{E}\left[g\left(z_{\mathrm{g}},\theta_{\mathrm{g},t}\right)^2\,\theta_{\mathrm{g},t}\right]\Big|_{\mathbf{z_m}=\mathbf{z}=\mathbf{z}^*} &= \overline{g^2}\,\overline{\theta} \\
\mathrm{E}\left[g\left(z_{\mathrm{g}},\theta_{\mathrm{g},t}\right)\,\theta_{\mathrm{g},t}^2\right]\Big|_{\mathbf{z_m}=\mathbf{z}=\mathbf{z}^*} &= \overline{g}\left(\overline{\theta}^2 + \frac{V\,\sigma_{\mathrm{g}}^2}{V+\sigma_{\mathrm{g}}^2}\right) \\
\mathrm{E}\left[g\left(z_{\mathrm{g}},\theta_{\mathrm{g},t}\right)^2\,\theta_{\mathrm{g},t}^2\right]\Big|_{\mathbf{z_m}=\mathbf{z}=\mathbf{z}^*} &= \overline{g^2}\left(\overline{\theta}^2 + \frac{V\,\sigma_{\mathrm{g}}^2}{2\,V+\sigma_{\mathrm{g}}^2}\right)
\end{aligned}
\tag{C15}
$$

and

$$
\begin{aligned}
\mathrm{E}\left[g\left(z_{\mathrm{g}},\theta_{\mathrm{g},t}\right)\,\theta_{\mathrm{f},t}\right]\Big|_{\mathbf{z_m}=\mathbf{z}=\mathbf{z}^*} &= \overline{g}\,\overline{\theta} \\
\mathrm{E}\left[g\left(z_{\mathrm{g}},\theta_{\mathrm{g},t}\right)^2\,\theta_{\mathrm{f},t}\right]\Big|_{\mathbf{z_m}=\mathbf{z}=\mathbf{z}^*} &= \overline{g^2}\,\overline{\theta} \\
\mathrm{E}\left[g\left(z_{\mathrm{g}},\theta_{\mathrm{g},t}\right)\,\theta_{\mathrm{f},t}^2\right]\Big|_{\mathbf{z_m}=\mathbf{z}=\mathbf{z}^*} &= \overline{g}\left(\overline{\theta}^2 + V\,\frac{V\left(1-\rho^2\right)+\sigma_{\mathrm{g}}^2}{\left(V+\sigma_{\mathrm{g}}^2\right)}\right) \\
\mathrm{E}\left[g\left(z_{\mathrm{g}},\theta_{\mathrm{g},t}\right)^2\,\theta_{\mathrm{f},t}^2\right]\Big|_{\mathbf{z_m}=\mathbf{z}=\mathbf{z}^*} &= \overline{g^2}\left(\overline{\theta}^2 + V\,\frac{2V\left(1-\rho^2\right)+\sigma_{\mathrm{g}}^2}{\left(2V+\sigma_{\mathrm{g}}^2\right)}\right) . \\
\mathrm{E}\left[g\left(z_{\mathrm{g}},\theta_{\mathrm{g},t}\right)\,\theta_{\mathrm{g},t}\,\theta_{\mathrm{f},t}\right]\Big|_{\mathbf{z_m}=\mathbf{z}=\mathbf{z}^*} &= \overline{g}\left(\overline{\theta}^2 + \rho\,\frac{V\,\sigma_{\mathrm{g}}^2}{V+\sigma_{\mathrm{g}}^2}\right) \\
\mathrm{E}\left[\theta_{\mathrm{g},t}\,\theta_{\mathrm{f},t}\right]\Big|_{\mathbf{z_m}=\mathbf{z}=\mathbf{z}^*} &= \overline{\theta}^2 + \rho\,V
\end{aligned}
\tag{C16}
$$

Putting eqs. (C14)-(C16) into eq. (C13) gives us the necessary components to analyze disruptive selection.

The entry $H_{\mathrm{gg}}(\mathbf{z}^*)$ of the Hessian matrix, that is the quadratic coefficient of selection on the germination trait, then becomes

$$
H_{\mathrm{gg}}\left(\mathbf{z}^*\right) = \frac{(1-s)}{\sigma_{\mathrm{g}}^4}\left(s\,V-\sigma_{\mathrm{g}}^2\right) \quad , \tag{C17}
$$

which is equal to $\mathrm{E}[H_2]$ in the single-trait case (eq. B16). This shows that selection on the germination trait alone is disruptive when $sV > \sigma_{\mathrm{g}}^2$, that is, when between-year environmental variance is large relative to the steepness of the germination reaction norm.

Similarly, the entry $H_{\mathrm{ff}}(\mathbf{z}^*)$ of the Hessian matrix, which is the quadratic coefficient of selection on the fecundity trait, is in fact

$$H_{\mathrm{ff}}\left(\mathbf{z}^*\right) = \frac{sV}{\sigma_{\mathrm{f}}^4}\left((1-s) - \overline{g^2}\, s\, \frac{\left(2V\left(1-\rho^2\right)+\sigma_{\mathrm{g}}^2\right)}{2V+\sigma_{\mathrm{g}}^2} - (1-2s)\,\overline{g}\,\frac{\left(V\left(1-\rho^2\right)+\sigma_{\mathrm{g}}^2\right)}{V+\sigma_{\mathrm{g}}^2} - \sigma_{\mathrm{f}}^2\,\frac{\left(1-\overline{\gamma}\right)}{Vs}\right), \quad \text{(C18)}$$

with $\overline{\gamma} = (1-\overline{g})\, s$, i.e. $\mathrm{E}[H_1]$ in the single-trait case (eq. B16). This expression shows that the strength and sign of disruptive selection on the fecundity trait depend not only on fecundity parameters such as the niche breadth σ_{f}^2, but also on germination plasticity through σ_{g}^2. As σ_{g}^2 decreases, the long-term germination probabilities $\overline{g}$ and $\overline{g^2}$ both decline (eq. C15), which increases generation overlap $\overline{\gamma}$ and modifies how variation in fecundity contributes to $H(z^*)$. Hence, greater germination plasticity can either increase or decrease H_{ff} depending on the balance between V, σ_{f}^2 and the induced changes in $\overline{g}$ and $\overline{g^2}$. In the limit of no germination plasticity ($\sigma_{\mathrm{g}}^2 \to \infty$), $H_{\mathrm{ff}}(\mathbf{z}^*)$ simplifies to

$$H_{\mathrm{ff}}\left(\mathbf{z}^*\right) = \frac{(1-(1-g_{\max})s)}{\sigma_{\mathrm{f}}^4}\left((1-g_{\max})sV - \sigma_{\mathrm{f}}^2\right),$$

recovering the classical branching condition $(1-g_{\max})s\,V > \sigma_{\mathrm{f}}^2$ (e.g., Chesson and Warner, 1981; Ellner and Hairston, 1994; Svardal et al., 2015).

Finally, the off-diagonal entry of the Hessian matrix, $H_{\mathrm{gf}}(\mathbf{z}^*)$, which is the coefficient of correlational selection on the germination and fecundity traits (Phillips and Arnold, 1989), is

$$H_{\mathrm{gf}}\left(\mathbf{z}^*\right) = \rho\, s\, V\,\frac{(1-s)V + \left(1-(1-\overline{g})s\right)\sigma_{\mathrm{g}}^2}{\sigma_{\mathrm{f}}^2\,\sigma_{\mathrm{g}}^2\,(V+\sigma_{\mathrm{g}}^2)}, \quad \text{(C19)}$$

which is equal to $\mathrm{E}[H_3]/2$ in the single-trait case (eq. B16). Since the fraction in eq. (C19) is strictly positive, the sign of $H_{\mathrm{gf}}(\mathbf{z}^*)$ is the sign of ρ. Thus, positive environmental correlation ($\rho > 0$) favours a positive covariance between z_{g} and z_{f}, whereas negative environmental correlation ($\rho < 0$) favours a negative covariance, as expected.

Importantly, irrespective of the sign of the environmental correlation ρ, increasing the magnitude of correlational selection (whether positive or negative) promotes polymorphism. This follows from the form of the leading eigenvalue of the Hessian matrix,

$$\lambda(\mathbf{z}^*) = \frac{1}{2}\left(H_{\mathrm{ff}}(\mathbf{z}^*) + H_{\mathrm{gg}}(\mathbf{z}^*) + \sqrt{(H_{\mathrm{ff}}(\mathbf{z}^*) - H_{\mathrm{gg}}(\mathbf{z}^*))^2 + 4H_{\mathrm{gf}}(\mathbf{z}^*)^2}\right), \quad \text{(C20)}$$

where the square-root term is strictly increasing in $H_{\mathrm{gf}}(\mathbf{z}^*)^2$. Increasing $|H_{\mathrm{gf}}(\mathbf{z}^*)|$, for instance through an increase in $|\rho|$, therefore increases $\lambda(\mathbf{z}^*)$ and hence the likelihood of evolutionary branching. Biologically, this reflects that when germination and fecundity can evolve jointly, selection favours an

association between z_g and z_f that perform well given the within-year association between germination and reproductive environments, especially when $|\rho|$ is large and early-season conditions strongly predict later conditions. See the main text for further interpretation and results.

D Supplementary Appendix - Simulations

To accompany our mathematical analyses of the *two-trait case*, we ran individual-based simulations in *R* for two separate scenarios: 1) clonally reproducing haploid plants, and 2) sexually reproducing diploid plants. We detail these simulations here, first describing the genetic architecture of traits in our simulations.

D.1 The genetic basis of traits

Haploid clonals

In simulations with haploid plants, the fecundity and the germination trait each was controlled by a separate genetic locus with individual allelic effect size $a_{\mathrm{g},i}$ and $a_{\mathrm{f},i}$, respectively. From these values, we computed the germination trait value for individual i as $z_{\mathrm{g},i} = a_{\mathrm{g},i}$ and the fecundity trait as $z_{\mathrm{f},i} = a_{\mathrm{f},i}$ (i.e., no pleiotropy, epistasis, or environmental trait contributions).

During reproduction, germinated plants produced clonal seeds that inherited the allelic effect sizes of their mother plant. For instance, germinated plant i with genotype $(a_{\mathrm{g},i},\ a_{\mathrm{f},i}) = (10.2,\ 9.9)$ produced seeds with genotype $(10.2,\ 9.9)$ where newly produced seeds then were subject to mutations (as detailed below).

Sexual diploids

In simulations with sexual diploid plants, the germination and fecundity trait each was controlled by a single locus with two homologous (maternal and paternal) copies, with the first copy $(a_{\mathrm{g},1,i},\ a_{\mathrm{f},1,i})$ and the second copy $(a_{\mathrm{g},2,i},\ a_{\mathrm{f},2,i})$. From these homologous copies, we computed the germination trait value $z_{\mathrm{g},i} = a_{\mathrm{g},1,i} + a_{\mathrm{g},2,i}$ and the fecundity trait value $z_{\mathrm{f},i} = a_{\mathrm{f},1,i} + a_{\mathrm{f},2,i}$.

During sexual reproduction, each germinated hermaphroditic plant produced haploid gametes that fused randomly with other gametes to form the next generation of diploid seeds. The genotypic composition of haploid gametes varied with the degree of genetic linkage between the germination and fecundity locus. In scenarios with complete genetic linkage, only the following two gametes were formed by individual i with equal probability: $(a_{\mathrm{g},1,i},\ a_{\mathrm{f},1,i})$ and $(a_{\mathrm{g},2,i},\ a_{\mathrm{f},2,i})$. In the absence of genetic linkage, individual i produced the following four gametes with equal probability: $(a_{\mathrm{g},1,i},\ a_{\mathrm{f},1,i})$, $(a_{\mathrm{g},2,i},\ a_{\mathrm{f},2,i})$, $(a_{\mathrm{g},1,i},\ a_{\mathrm{f},2,i})$, $(a_{\mathrm{g},2,i},\ a_{\mathrm{f},1,i})$.

Mutations

Mutations occurred randomly in newly produced seeds with a per-locus-and-copy mutation rate $\mu = 0.00025$ in diploids (and a per-locus mutation rate of $\mu = 0.0005$ in haploids), at each locus separately. The mutational effects were picked from a normal distribution $\mathcal{N}_1\,(0,0.01)$ and added to the existing allelic value following the continuum-of-alleles model.

Initial genetic composition

At the beginning of each simulation run, the allelic effect sizes for each copy $a_{\mathrm{g},i}$ and $a_{\mathrm{f},i}$ were picked from a normal distribution $\mathcal{N}_1(5, 0.25)$ for each diploid individual separately (with $\mathcal{N}_1(10, 0.25)$ for $a_{\mathrm{g},i}$ and $a_{\mathrm{f},i}$ in the haploid case).

D.2 Life-Cycle Events

The life cycle events in the simulations follow exactly those described in the Model section of the main text.

0) Environmental variation We picked the environmental conditions during germination and reproduction $\theta_{\mathrm{g},t}$ and $\theta_{\mathrm{f},t}$ each year t anew from a bivariate normal distribution

$$\boldsymbol{\theta}_t \sim \mathcal{N}_2\left(\boldsymbol{\mu}, \boldsymbol{\Sigma}\right) \tag{D1}$$

with the vector of average environmental conditions

$$\boldsymbol{\mu} = \begin{pmatrix} 10 \\ 10 \end{pmatrix} \tag{D2}$$

and environmental variance-covariance matrix

$$\boldsymbol{\Sigma} = \begin{pmatrix} V & \rho V \\ \rho V & V \end{pmatrix} . \tag{D3}$$

1) Seed germination At the beginning of each year, each of the N seeds in the seed bank germinated with germination probability $g\left(z_{\mathrm{g},i}, \theta_{\mathrm{g},t}\right)$ following eq. (1). Each non-germinated seed survived in the seed bank to the next year with probability s and died with probability $1 - s$. Germination and death of seeds leave a total of $0 \leq N_{\mathrm{new},t} \leq N$ spots open in the seed bank to be filled.

2) Plant growth, reproduction, and regulation With clonal reproduction, we sampled $N_{\text{new},t}$ new haploid seeds from the genotypes of germinated plants where the weights were given by fecundity $f\left(z_{\text{f},i}, \theta_{\text{f},t}\right)$ following eq. (2). The number of newly produced seeds $N_{\text{new},t}$ was adapted each year anew to replenish the seedbank to a total of N seeds at the end of each year.

With sexual reproduction in diploids, we sampled $2N_{\text{new},t}$ haploid gametes with weights given by $f\left(z_{\text{f},i}, \theta_{\text{f},t}\right)$ and these haploid gametes fused randomly with other gametes to form new diploid seeds.

Newly produced seeds were subject to mutations as described above.

D.3 Simulation setting and summary statistics

We ran each simulation for T=20 000 years with a total seed number of N=10 000 and 20 replicates per parameter combination. At evolutionary equilibrium, we computed the total (long-term) phenotypic variance at the germination and fecundity trait for the last 500 years with a 25-year sample interval ($t = (19500, 19525, 19550, ..., 20000)$)

$$\begin{aligned} P_{\text{g,tot}} &= \frac{1}{n_T} \sum_{t=19500}^{T} \frac{1}{N_{\text{g},t}} \sum_{i=1}^{N_{\text{g},t}} \left(z_{\text{g},i} - \overline{z}_{\text{g}}\right)^2 \\ P_{\text{f,tot}} &= \frac{1}{n_T} \sum_{t=19500}^{T} \frac{1}{N_{\text{f},t}} \sum_{i=1}^{N_{\text{f},t}} \left(z_{\text{f},i} - \overline{z}_{\text{f}}\right)^2 \end{aligned} \tag{D4}$$

for $n_T = 21$ sampled years, with the long-term average trait value $\overline{z}_{\text{g}} = \frac{1}{n_T} \sum_{t=19500}^{n_T} \frac{1}{N_{\text{g},t}} \sum_{i=1}^{N_{\text{g},t}} \left(z_{\text{g},i}\right)$ and the number of germinated plants in year t being $N_{\text{g},t}$.

We further partitioned this total phenotypic variance into two variance components, $P_{\text{g,tot}} = P_{\text{g,w}} + P_{\text{g,b}}$ and $P_{\text{f,tot}} = P_{\text{f,w}} + P_{\text{f,b}}$. First, we computed within-year phenotypic variance

$$\begin{aligned} P_{\text{g,w}} &= \frac{1}{n_T} \sum_{t=19500}^{T} \frac{1}{N_{\text{g},t}} \sum_{i=1}^{N_{\text{g},t}} \left(z_{\text{g},i} - \overline{z}_{\text{g},t}\right)^2 \\ P_{\text{f,w}} &= \frac{1}{n_T} \sum_{t=19500}^{T} \frac{1}{N_{\text{f},t}} \sum_{i=1}^{N_{\text{f},t}} \left(z_{\text{f},i} - \overline{z}_{\text{f,t}}\right)^2 \end{aligned} \tag{D5}$$

with the average trait value in year t being $\overline{z}_{\text{g},t}$. Second, we calculated between-year phenotypic variance

$$\begin{aligned} P_{\text{g,b}} &= \frac{1}{n_T} \sum_{t=19500}^{T} \left(\overline{z}_{\text{g},t} - \overline{z}_{\text{g}}\right)^2 \\ P_{\text{f,b}} &= \frac{1}{n_T} \sum_{t=19500}^{T} \left(\overline{z}_{\text{f,t}} - \overline{z}_{\text{f}}\right)^2 \end{aligned} . \tag{D6}$$

Finally, we computed the phenotypic covariance between the germination and fecundity trait within years

$$P_{\mathrm{gf,w}} = \frac{1}{n_T} \sum_{t=19500}^{n_T} \frac{1}{N_{\mathrm{g},t}} \sum_{i=1}^{N_{g,t}} \left(z_{\mathrm{g},i} - \overline{z}_{\mathrm{g},t}\right)\left(z_{\mathrm{f},i} - \overline{z}_{\mathrm{f,t}}\right) \ . \tag{D7}$$

D.4 Simulations with fluctuating seed densities

To relax the assumption of a constant seed-bank size, we ran individual-based simulations with variable N_t similar to the model of Kortessis and Chesson (2021). In these simulations, we assumed competition among reproducing plants with the following life-cycle events:

Seed germination

Each seed indexed i in year t germinated with probability $g\left(z_{\mathrm{g},i}, \theta_{\mathrm{g},t}\right)$ as in the model of the main text (eq. 1). Each non-germinated seed survived to the next year with probability s and died with probability $1 - s$. As a consequence of stochastic seed germination and survival, the number of non-germinated seeds that survived to the next year varied among years.

Plant growth, reproduction, and regulation

With clonal reproduction, we sampled N_{new} new haploid seeds from the genotypes of germinated plants with weight

$$f\left(z_{\mathrm{f},i}, \theta_{\mathrm{f},t}\right) = \frac{\mathrm{Exp}\left[-\frac{\left(z_{\mathrm{f},i} - \theta_{\mathrm{f},t}\right)^2}{2\,\sigma_{\mathrm{f}}^2}\right]}{\sum_{j=1}^{N_{\mathrm{germ}}} \mathrm{Exp}\left[-\frac{\left(z_{\mathrm{f},j} - \theta_{\mathrm{f},t}\right)^2}{2\,\sigma_{\mathrm{f}}^2}\right]} \ , \tag{D8}$$

where N_{germ} denotes the number of germinated plants. The number of newly produced seeds N_{new} was constant among years, but chosen such that the seed bank at the end of each year was $N_t \approx 10\,000$.

With sexual reproduction in diploids, we sampled $2N_{\mathrm{new}}$ haploid gametes with weights given by eq. (D8) and these haploid gametes then fused randomly to form new diploid seeds.

Newly produced seeds were subject to mutations as described above.

Results

Figure D1 shows simulation results for the two-trait case with clonal reproduction in haploid plants, allowing the size of the seed bank to vary among years. Density dependence among germinated plants was implemented through the fecundity function (D8), such that a variable number of seeds germinated each year and produced a total of N_{new} new seeds, all of which entered the seed bank. As a result, seed-bank size fluctuated over time. The outcomes are nearly identical to those shown in Fig. 4 of the main text, indicating that predictive germination continues to strengthen the storage effect even when seed numbers vary across years, at least when competition occurs among plants for reproduction.

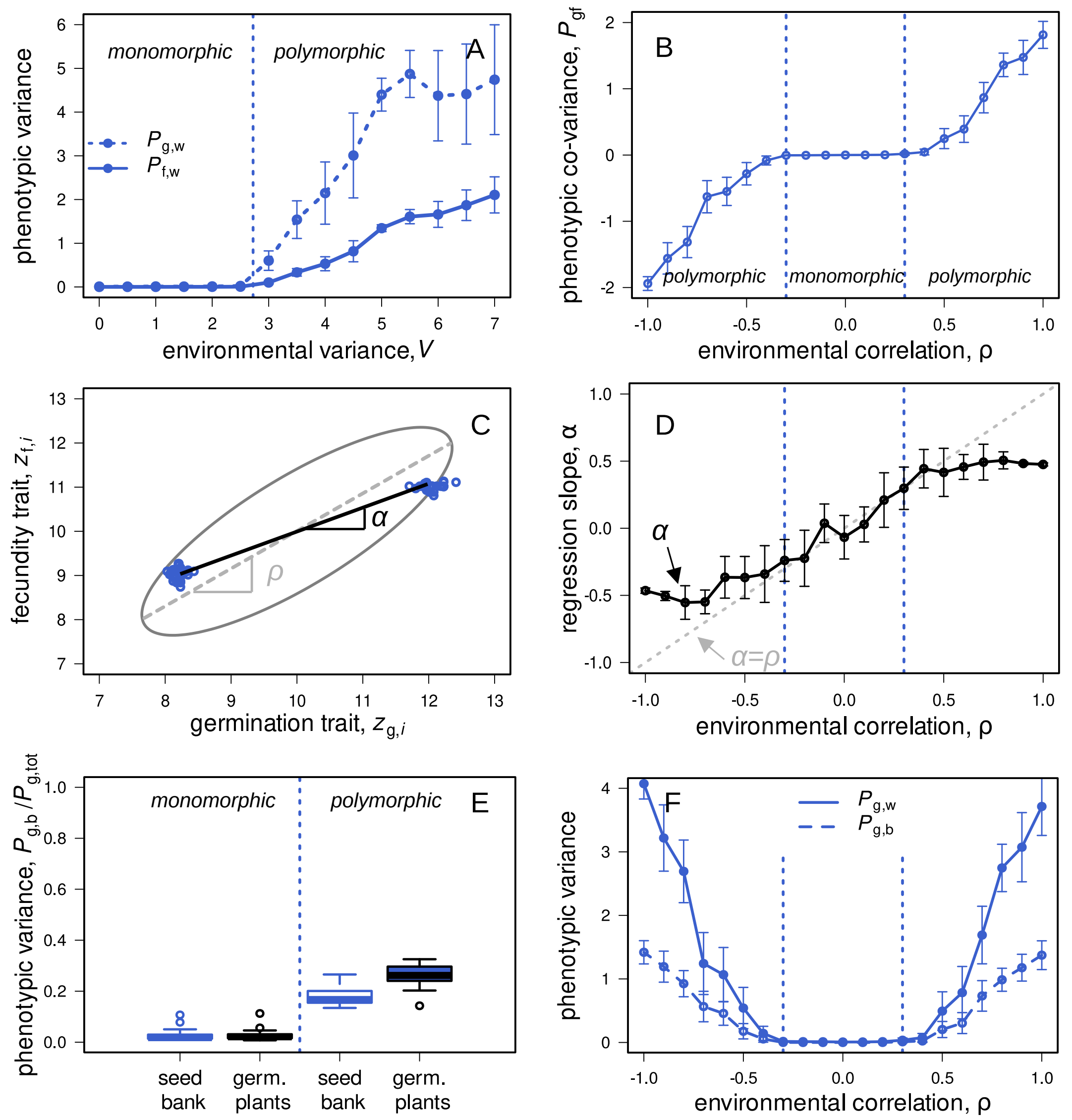

Figure D1: Graph **A** depicts within-year phenotypic variance in the germination trait (dashed lines) and the fecundity trait (solid lines) as a function of interannual environmental variation V. The vertical dotted lines (—) present the threshold between stabilizing and disruptive selection as predicted by the mathematical model (following the Hessian matrix C6 with components C17-C19). Graph **B** illustrates the extent of trait covariance P_{gf} (see eq. D7) as a function of environmental correlation, where polymorphism could evolve with positive and negative ρ. Graph **C** conceptualizes the linear regression slope for the phenotypic distribution (α) and for the environmental distribution (ρ), where plants not always evolve $\alpha = \rho$ (graph **D**). Graph **E** shows simulation results of the between-year phenotypic variance ($P_{g,b}/P_{g,tot}$) for seeds in the seed bank and for germinated plants only. Graph **F** presents the between-year phenotypic variance as a function of environmental correlation. The parameter values for graphs A-F are: V=4, g_{max}=0.5, s=0.8, σ_f^2=2, σ_g^2=10 (with ρ=0.8 for graph A and C; and ρ=(0,1) for graph E). The total number of newly produced seeds f_{max} was picked such that the total number of seeds at the end of each year was $N \approx 10\,000$ (while showing variation among years). The whiskers in graphs A,B,D,F illustrate variation among replicates (covering one standard deviation).

References

Chesson, P. L. (1983). Coexistence of Competitors in a Stochastic Environment: The Storage Effect. In Levin, S., Freedman, H. I., and Strobeck, C., editors, Population Biology, volume 52, pages 188–198. Springer Berlin Heidelberg, Berlin, Heidelberg. Series Title: Lecture Notes in Biomathematics.

Chesson, P. L. and Warner, R. R. (1981). Environmental variability promotes coexistence in lottery competitive systems. The American Naturalist, 117(6):923–943.

Ellner, S. P. and Hairston, N. G. (1994). Role of overlapping generations in maintaining genetic variation in a fluctuating environment. The American Naturalist, 143(3):403–417.

Geritz, S. A. H., Kisdi, E., Meszéna, G., and Metz, J. A. J. (1998). Evolutionary singular strategies and the adaptive growth and branching of the evolutionary tree. Evolutionary Ecology, 12:35–57.

Kortessis, N. and Chesson, P. (2021). Character displacement in the presence of multiple trait differences: Evolution of the storage effect in germination and growth. Theoretical Population Biology, 140:54–66.

Kotz, S., Balakrishnan, N., and Johnson, N. L. (2000). Continuous Multivariate Distributions: Models and Applications. Wiley Series in Probability and Statistics. Wiley.

Leimar, O. (2009). Multidimensional convergence stability. Evolutionary Ecology Research, 11:191–208.

Lewontin, R. C. and Cohen, D. (1969). On population growth in a randomly varying environment. Proceedings of the National Academy of Sciences, 62(4):1056–1060.

Phillips, P. C. and Arnold, S. J. (1989). Visualizing multivariate selection. Evolution, 43(6):1209–1222.

Svardal, H., Rueffler, C., and Hermisson, J. (2011). Comparing environmental and genetic variance as adaptive response to fluctuating selection. Evolution, 65(9):2492–2513.

Svardal, H., Rueffler, C., and Hermisson, J. (2015). A general condition for adaptive genetic polymorphism in temporally and spatially heterogeneous environments. Theoretical Population Biology, 99:76–97.

Sæther, B.-E. and Engen, S. (2015). The concept of fitness in fluctuating environments. Trends in Ecology and Evolution, 30(5):273–281.
